# Biochemical Pathways of Cellular Mechanosensing/Mechanotransduction and Their Role in Neurodegenerative Diseases Pathogenesis

**DOI:** 10.3390/cells11193093

**Published:** 2022-10-01

**Authors:** Ilaria Tortorella, Chiara Argentati, Carla Emiliani, Francesco Morena, Sabata Martino

**Affiliations:** 1 Department of Chemistry, Biology and Biotechnology, University of Perugia, Via del Giochetto, 06123 Perugia, Italy; 2 Centro di Eccellenza CEMIN (Materiali Innovativi Nanostrutturali per Applicazioni Chimica Fisiche e Biomediche), University of Perugia, 06123 Perugia, Italy

**Keywords:** mechanosensing, mechanotransduction, neurodegenerative diseases, mechanobiology, Alzheimer’s Disease, Huntington’s Disease, Amyotrophic Lateral Sclerosis, Parkinson’s Disease

## Abstract

In this review, we shed light on recent advances regarding the characterization of biochemical pathways of cellular mechanosensing and mechanotransduction with particular attention to their role in neurodegenerative disease pathogenesis. While the mechanistic components of these pathways are mostly uncovered today, the crosstalk between mechanical forces and soluble intracellular signaling is still not fully elucidated. Here, we recapitulate the general concepts of mechanobiology and the mechanisms that govern the mechanosensing and mechanotransduction processes, and we examine the crosstalk between mechanical stimuli and intracellular biochemical response, highlighting their effect on cellular organelles’ homeostasis and dysfunction. In particular, we discuss the current knowledge about the translation of mechanosignaling into biochemical signaling, focusing on those diseases that encompass metabolic accumulation of mutant proteins and have as primary characteristics the formation of pathological intracellular aggregates, such as Alzheimer’s Disease, Huntington’s Disease, Amyotrophic Lateral Sclerosis and Parkinson’s Disease. Overall, recent findings elucidate how mechanosensing and mechanotransduction pathways may be crucial to understand the pathogenic mechanisms underlying neurodegenerative diseases and emphasize the importance of these pathways for identifying potential therapeutic targets.

## 1. Introduction

In recent years, many works have explored the molecular basis of mechanobiology to clarify how mechanosensing and mechanotransduction pathways guide cells to (i) sense their microenvironment physicochemical properties and respond accordingly, and (ii) modify their surroundings transmitting specific mechanical information extracellularly via inside/outside signaling routes. While these pathways’ mechanistic components are mostly uncovered today [1,2], the tight interconnection between mechanosignaling and biochemical signaling of soluble molecules involved in crucial cellular pathways (i.e., cellular differentiation, proliferation, cell death) has been brought to light only in recent years. Since then, many studies have focused on understanding the mechanisms underlying these processes, with particular attention to diseased systems, to pave the way for new frontiers in drug targeting and therapy development [3,4]. The development of in vitro systems that could faithfully resemble the in vivo phenomena that occur when cells and tissues are subjected to mechanical alteration is one of the fastest developing research areas, with many new cell modeling strategies such as 3D stem cell cultures and biomaterials, used for building fundamental tools for disease modeling [5]. It has been demonstrated that modulating the physical and chemical features of biomaterials, including surface morphology, topography, wettability and architecture, allows to tune and understand the biological response of cells [6]. Using biomaterials of different origins enables not only eliciting specific cell differentiation programs for tissue engineering applications, but also to study mechanotransduction and mechanosensing signaling occurring in response to a specific altering of the external environment [7,8,9,10,11,12]. Parallell to the development of more accurate in vitro disease models, efforts are being made for the in vivo study of mechanics in healthy and diseased systems, but limited studies are available because of their complexity [13,14,15,16,17]. The importance of tissue mechanics has already been established for some diseases where the relationship between mechanical environment and disease pathogenesis is more straightforward [18]. Cancer malignancy and invasive potential [19,20,21,22,23], fibrosis [24,25,26], vascular-related diseases [27,28,29,30] and musculoskeletal disorders [31,32,33,34,35], are the pathologies for which the association of pathological features and impairment of the mechanical characteristics of diseased tissues/cells has been proven and quite well-characterized. This association is, however, crucial for the development of malignancies in every tissue, and the brain is one of them, as the regulation of its elastic properties is fundamental for its functioning and homeostasis [36]. Among the events that participate in neurodegeneration, alterations of the cerebral and CNS mechanical microenvironment are now recognized as important contributing factors. Therefore, to better comprehend the pathological molecular changes that take place through the progression of neurodegenerative diseases, it is fundamental to understand the association between mechanical changes and biochemical signaling occurring in their pathogenesis [37,38].

To this end, in this review we recapitulate the current knowledge of the molecular mechanisms underlying mechanosensing and mechanotransduction, including recent advances about how these pathways establish crosstalk with intracellular organelles, actively participating in their homeostasis and dysfunction. Hence, we discuss how all these mechanisms converge towards the identification of interconnections between soluble molecular signaling and mechanical stimuli that contributes to the main neurodegenerative diseases, i.e., Alzheimer’s Disease (AD), Huntington’s Disease (HD), Amyotrophic Lateral Sclerosis (ALS) and Parkinson’s Disease (PD).

## 2. Mechanobiology—A Brief Overview

Mechanobiology defines how cells generate, perceive, decode and adjust to physical cues at the molecular level, and describes how cellular components (often referred to as mechanosensors) participate in the propagation of intracellular mechanical signals that culminate in the activation of a biological response [39]. The propagation of mechanical forces is considered to be one of the fastest signaling routes, faster than passive diffusion and molecular transport, therefore enabling a fast translation of physical stimuli into a metabolic response, which explains cells’ ability to quickly adjust to constantly evolving surroundings [40]. Part of the explanation of these phenomena derives from the biological application of the architectural concept of “Tensegrity” first introduced by Donald Ingber, which describes the tensional integrity of cells in a state of prestress, which allows them to instantly respond to mechanical stresses [41,42]. Indeed, it is now commonly accepted that whole organs, tissues and cells behave as hierarchical tensegrities in which, when a perturbation in the force occurs, all elements transmit the stress from the macro- to the microscale across load-carrying components [43].

The maintenance of the cell’s mechanical homeostasis is a fine balance that relies on the control of cellular density, and investigations of the rheological characteristics of the cytoplasm have found that they differ depending on which cellular site is considered. Typically, the nuclear and perinuclear areas are stiffer compared to the periphery of the cell because of their difference in composition; cellular regions with a higher concentration of cytoskeletal or soluble proteins are usually stiffer, as well as cells with reduced volume due to the formation of intracellular crowding [44]. Although much research is being done to characterize the role of mechanotransducers and discover new cellular elements involved in the sensing and propagation of mechanical signaling, it is important to remember that, ultimately, the cell as a whole entity can be considered as a mechanosensor [43]. The type of response to certain mechanical stimuli is influenced by a variety of factors, including cell type, shape and the elaborate tuning of highly complex dynamic signaling networks which can result in a range of cell fate decisions depending on the type and timing of perturbation [43]. As a general principle, this response can be ascribed to the adjustment of cellular viscoelasticity, primarily based on the dynamic interactions of the main cytoskeletal components, Actin fibers, microtubules and intermediate filaments [45,46]. These three components organize with each other thanks to cross-linking and motor proteins, resulting in the formation of bundles and networks spanning from more rigid (rods) to flexible (coils) structures that confer to cells characteristic mechanical properties; the adaptation to different mechanical stimuli thus relies on the adjustment of these complex cytoskeletal networks, rather than on the individual response of a single component [46]. Eventually, forces are perceived by the cell, and the generated tension is propagated throughout the cytoskeleton up to the nucleus, where mechanical stimuli are translated into changes in gene expression programs to stimulate certain cell functions [47]. This response is the result of two complex and interrelated signaling pathways known as “mechanosensing” and “mechanotransduction”: mechanosensing refers to the ability to perceive changes in the mechanical environment in which cells are, whereas the term mechanotransduction is used to include all the molecular events that convert changes in extracellular forces into soluble biochemical signaling that stimulates specific cell functions, as well as the events that lead to the generation of cellular forces by which cells adjust their microenvironment’s features [39]. These two processes operate concomitantly to gather all the extra- and intra-cellular mechanical signals; the principal axes that mediate cellular response to mechanical stimuli pass through (i) the extracellular matrix (ECM)-focal adhesions (FAs) complexes and ECM-integrins, then to the cyto- and nucleo-skeleton, or (ii) adherens junctions (AJs) complexes that mediate cell to cell contacts and transmit information intercellularly, which then propagates across the cytoskeleton and arrives to the nucleus. With these two mechanisms, cells are able to sense the surrounding environment and remodel their microenvironment to affect adjacent cells and steer tissues and organs’ fates [5]. Although a detailed explanation of these phenomena would require an extensive analysis that we have previously provided elsewhere [39], we will highlight the essential characteristics of the mechanosensing and mechanotransduction pathways in the following sections.

### 2.1. Mechanosensing

In the past decades, much research has been done to characterize the different mechanical environments existing in human tissues; however, no model has proven to be sufficiently comprehensive to explain the complexity of forces balance required to maintain cellular homeostasis [48].

**Figure 1:**
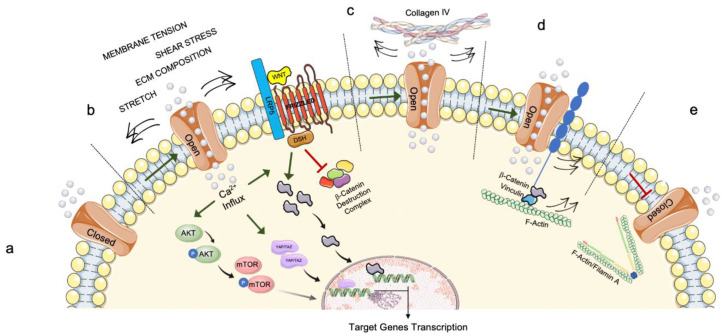
Schematic representation of mechanical regulation of Piezo channels: (**a**) When no stimuli are present, Piezo channels are closed and inhibit the passage of ions; (**b**) Different mechanical cues stimulate Piezo opening through forces that are conveyed by the stretching of the lipid bilayer and allow the entering of positive ions such as Calcium inside the cell where it triggers pathways such as Akt/mTOR, YAP/TAZ and Wnt/β-Catenin, allowing the transcription of target genes; (**c**) Collagen IV presence in the ECM induces Piezo opening; (**d**) Piezo interacts with the E-Cadherin/β-Catenin/F-Actin complex which generates intracellular forces that favor the channels’ opening; (**e**) Piezo interaction with Filamin A is desensitizing and inhibits its opening. AKT, RAC-alpha serine/threonine-protein kinase; Ca^2+^, Calcium ion; DSH, Dishevelled; ECM, extracellular matrix; LRP5, LDL Receptor Related Protein; mTOR, Mammalian target of rapamycin; TAZ, transcriptional co-activator with PDZ-binding motif; WNT, Wingless/Integrated; YAP, Yes-associated protein 1.

The sensing of forces applied from the extracellular environment is one of the earliest events that occurs when cells respond to mechanical stimuli. Among these forces, the most studied as key factors for stem cells differentiation and reinforcement of pathogenic states (e.g., in cancer cells) are (i) hydrostatic pressure exerted omnidirectionally by fluids surrounding the cells membrane [49]; (ii) shear stress generated when fluids act tangentially on cells surfaces [39]; (iii) membrane tension, which is closely connected with mechanosensitive channels opening and can be simplified as two opposing forces pulling cells to opposite directions causing their elongation [49]; (iv) compression that leads to cellular contraction; and, the two opposing mechanical properties describing the dynamic response of the cells to different forces, (v) stiffness, expressing cellular resistance to deformation, and (vi) elasticity, or cells’ ability to return to their original state after being subjected to a force [5]. All these forces are sensed by cells in their entirety, as the process of mechanical sensing involves a number of proteins denominated ‘mechanosensors’ for their characteristic conformational change when subjected to force application, which results in the opening/activation of an active site mediating a certain biochemical function [50,51]. In other words, localized force variations are applied to cell membranes’ proteins and these mechanical stimuli can directly activate mechanosensitive ion channels that induce changes in intracellular signaling [47]; it has been shown that the activation of mechanically activated (MA)-ion channels represents the fundamental step for cellular mechanotransduction [50,52]. MA-ion channels gating is responsive to both tension and shear stress occurring at the cell membrane, and to direct cell–cell contact and intracellular cytoskeletal rearrangements [50]. Among the MA-ion channels, the Piezo proteins play a key role in transmitting information from the extracellular environment about the ECM’s variation in forces and topography. Since their initial description in 2010, the significance of these proteins has become increasingly clear, with particular attention to Piezo1 and Piezo2 as essential mediators of bone differentiation, skeletal cells mechanotransduction, and mesenchymal stem cells differentiation [52]. Furthermore, Piezo proteins have been identified as key modulators of neurogenesis and neural stem cells’ commitment [53,54], and their mutations have been linked to the onset of various pathologies, such as lymphatic dysplasia and other diseases characterized by dysfunctional mechanosensory neurons [55,56]. All this, along with the recent discovery of Piezos’ role in CNS correct myelination [57], supports the idea that these channels are important for the maintenance of neural homeostasis and suggests that they might have a role in neurodegenerative disease pathogenesis; despite the fact that today there are currently no conclusive links between Piezo gene mutations and the onset of a particular neurodegenerative disorder, several studies have linked Piezo channel dysfunctions and subsequent downstream activation signaling deregulation to diseases such as AD, Multiple Sclerosis, Dementia and Type-2 Diabetes [58,59,60]. These channels can be gated by the cells in response to mechanical cues enabling extracellular positively charged ions, mainly Calcium ions, to pass through the cell membrane and stimulate intracellular responses by triggering numerous pathways such as Yes-associated protein 1 (YAP1), Wnt/β-Catenin, and Akt mediated signaling (Figure 1a,b) [50,52,61]. The tight relationship between Piezo channels and their mechanical environment results in their modulation by interactions with the ECM and the cytoskeletal components. For example, Collagen IV presence in the ECM sensitizes Piezo channels (Figure 1c), while their interaction with Filamin A seems to be desensitizing, as loss of Filamin A activates them (Figure 1e) [62,63,64,65]. Similarly, a recent study highlighted that Piezo1 intimately interacts with the E-Cadherin/β-Catenin/F-Actin complex and that removal of either E-Cadherin or β-Catenin results in channels’ desensitization (Figure 1d) [66].

Another family of channels that transform mechanical stress into electrochemical signaling is the TREK family (also known as Potassium channel subfamily K member 2), responsible for the entrance of potassium currents in cells, and which can be found in both peripheral and central nervous systems [47]. In particular, three members of this family (TREK-1, TREK-2 and TRAAK) are activated by mechanical stimuli such as cellular deformation, swelling and membrane stretch [67]. Notably, TREK-1 currents may play a role in mediating the cardiac mechanoelectrical balance; under pathological circumstances, such as aberrant cardiac muscle stretch or cellular swelling caused by ischemia, these currents could be involved in cardiac excitability deregulation [67].

### 2.2. Mechanotransduction

**Figure 2:**
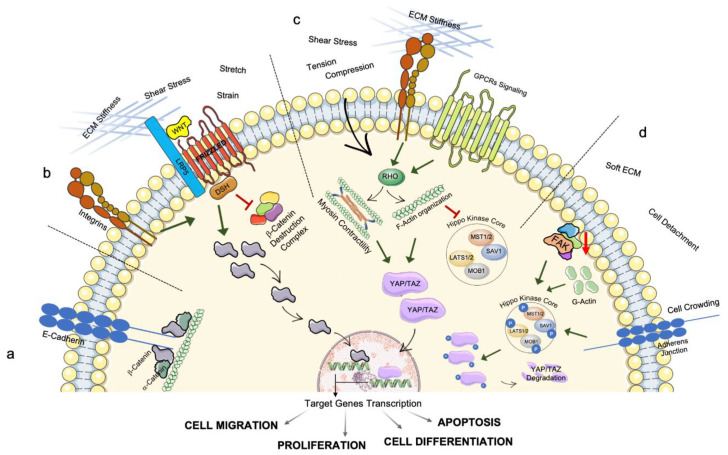
Schematic representation of the principal biochemical pathways that mediate mechanotransduction: (**a**) β-Catenin at the membrane level binds E-Cadherin and contributes to cellular adhesion; (**b**) Activation of the Wnt/ β-Catenin pathway: Wnt ligands and mechanical stimuli mediated by integrins inhibit the β-Catenin destruction complex and induce β-Catenin translocation to the nucleus where its leads to transcription of target genes involved in diverse cellular functions; (**c**) Activation of YAP/TAZ nuclear translocation and transcriptional co-activator function is induced by ECM mechanical properties and external mechanical stimuli, which are sensed by the cell through the integrins and GPCRs signaling routes; with the mediation of the RHO GTPase pathway, F-Actin formation and contractility through Myosin II induce YAP/TAZ nuclear translocation and inactivation of the Hippo kinase core, allowing transcription of target genes; (**d**) Mechanical stimuli such as cellular detachment, soft ECM, together with excessive formation of adherens junction due to cell crowding and inhibition of FAK or F-Actin formation, cause YAP/TAZ inhibition due to phosphorylation of the core Hippo kinases and of YAP/TAZ, leading to their cytoplasmatic degradation. Red arrow down ↓ indicates downregulation. DSH, Dishevelled; FAK, Focal Adhesion Kinase; LATS1/2, large tumor suppressor kinase 1 and 2; LRP5, LDL Receptor Related Protein; MOB, MOB kinase activator 1; MST1, Ste20-like kinase 1; RHO, Ras homologous; SAV1, Salvador homolog 1; TAZ, transcriptional co-activator with PDZ-binding motif; WNT, Wingless/Integrated; YAP, Yes-associated protein 1.

Today it is widely accepted that mechanical signaling is a type of biological crosstalk that goes hand in hand with the better-known biochemical signaling [68]. Despite recent efforts to learn more about how mechanotransduction is used to fine-tune cell and tissue functioning in a variety of conditions, the specific regulatory mechanisms are still mostly unclear [69]. Nevertheless, the general mechanisms by which mechanical cues are translated into biochemical signals are well-described and have the common feature of involving proteins’ structural change and their shuttling from their original site to a new one where they fulfill their signaling function [70]. The majority of these mechanisms regard the outside–inside signaling axis, which encompasses the sensing of external mechanical stimuli, their cytoskeletal propagation and the shuttling of membrane or cytoskeletal proteins to the nucleus, where they activate the expression or inhibition of certain gene patterns. For example, β-Catenin, the central protein of the Wnt pathway, can be found in different cellular districts depending on its function: at the membrane level, it binds E-Cadherin and contributes to cellular adhesion, while when the Wnt pathway is active, β-Catenin is translocated to the nucleus where it acts as transcriptional co-activator for many different genes regulating important cellular functions such as proliferation, migration, apoptosis and differentiation (Figure 2a,b) [71,72,73]. One key aspect of this pathway is that it can be regulated by mechanical inputs, in addition to Wnt ligands, as it was proposed that mechanoactivation of Wnt/β-catenin signaling could be mediated by integrins, ECM stiffness, membrane stretch, strain and shear stress (Figure 2b) [71,74].

Likewise, some FAs proteins show nuclear localization depending on the mechanical environment, even though the explanation of this phenomenon is still not clearly understood; recently, both Paxillin and Talin 1 have been described as interacting with the nucleus with their specific domains, where they are thought to have a role in mRNA trafficking and genome stabilization [75,76,77].

A more well-described pathway tightly interrelated with the transduction of mechanical stimuli is the Hippo pathway and the activity of its transcriptional co-activators YAP and TAZ (Transcriptional co-activator with PDZ-binding motif). The Hippo pathway kinase core is composed of Ste20-like kinase 1 (MST1) associated with the cofactor Salvador homolog 1 (SAV1), and of large tumor suppressor kinase 1 and 2 (LATS1/2) and MOB kinase activator 1 (MOB1). When this core is active, YAP/TAZ are phosphorylated, their transcriptional co-activator role is inhibited, and they are exported from the nucleus to the cytoplasm, where they are degraded (Figure 2d). When the Hippo core is inactivated, YAP/TAZ is dephosphorylated and translocate from the cytoplasm to the nucleus, where they interact with DNA-binding proteins of the TEAD family and contribute to the expression of genetic programs involved in cell migration, survival, proliferation, and stem cells renewal or differentiation (Figure 2c,d) [78,79]. Among the stimuli that regulate this pathway, different mechanical cues have the ability to activate/inhibit YAP/TAZ nuclear translocation, including changes in the environmental biophysical properties (ECM stiffening and micropatterns), flow disturbance and shear stress, cell crowding, cell detachment as well as cell spreading or, more generally, changes in the expression of mechanosensing/mechanotransduction proteins (Figure 2c) [69,80]. For example, Focal Adhesion Kinase (FAK) or F-Actin formation inhibition has been shown to cause YAP/TAZ inactivation, therefore the monitoring of this pathway is central to understanding the role of mechanical impairments in diseased systems (Figure 2c,d) [80]. Moreover, YAP/TAZ responds to the Ras homolog family member A (RhoA) GTPase signaling pathway, another signaling pathway essential in mechanotransduction. Rho proteins regulate F-Actin organization and contractility through regulation of Myosin II activity, as well as intracellular force generation, cell proliferation and differentiation [81]. It has been shown that mechanical tension and compressive force are able to activate Rho activity and transduce these forces to mediate cellular response (Figure 2c) [80,81,82,83], and it was suggested that YAP/TAZ regulation of autophagy is a type of checkpoint that ensures the fulfillment of cytoplasmic remodeling and could be seen as a sort of epigenetic memory mechanism for maintaining cell differentiation [84].

## 3. Intracellular Transmission of Mechanosensing and Mechanotransduction Pathways: The Involvement of Organelles’ Homeostasis and Dysfunction

Although much research has been done to characterize how the external mechanical microenvironment influences cells’ homeostasis, the events occurring when mechanical stress originates intracellularly are less clear. That is the case of many neurodegenerative disorders in which misfolded proteins tend to self-assemble into pathological inclusions that lead to the liquid–liquid phase separation phenomenon that influences cells’ mechanical properties [85,86,87,88,89]. The association between aggregates and cytoskeletal proteins is a vicious cycle in which protein aggregation induces cytoskeletal destabilization, and in turn, the cytoskeleton’s alteration favors the progressive accumulation of pathological inclusions. For instance, the intermediate filaments protein Vimentin has a central role in spatially regulating the aggresome (containing misfolded/damaged proteins) to interact with proteasomes and favor proteostasis, fundamental in protein quality control and cells’ correct cell cycle progression [90].

The mechanical signaling occurring in the cytoskeleton ultimately leads to the regulation of the organization and functioning of cytoplasmatic components, especially cellular organelles. Cellular organelles in the cytoplasm are organized in a dynamical and elaborate manner that is not just the consequence of arbitrary interaction; through cytoskeletal transport via F-Actin filaments and microtubules, organelles migrate to specific locations inside cells to maintain cell homeostasis, but when mechanical alterations exceed a physiological limit, these balanced mechanisms are impaired [91]. Additionally, cytoskeletal elements can impose mechanical stress on organelles during their dynamic polymerization–depolymerization activities. As a result, organelles are continually subjected to tension and compression forces and therefore have to respond accordingly to maintain their correct functioning. However, the type of responses that organelles enact is still mostly unclear, particularly regarding the Endoplasmatic Reticulum (ER) and the Golgi Apparatus, for which only a few studies are available, whereas more information has been gathered on mitochondria and lysosomes, together with the autophagic and endo-lysosomal system, as they have a central role in cellular metabolic homeostasis [92,93,94]. Recent findings on the crosstalk between mechanical stimuli and the biochemical response of cellular organelles in homeostasis and dysfunction are presented in Table 1.

Mitochondria are fundamental cellular organelles for many metabolic cellular processes and their dysregulation is becoming a more prominent interesting mechanism in the study of degenerative disorders development [123]. Mechanotransduction signaling can alter the mitochondria ability to provide energy to cells with Adenosine Triphosphate (ATP), regulate apoptosis and participate in Ca balancing and ROS formation (Table 1) [100]. That is because, in order to fulfill these functions, mitochondria organize in a network that moves across microtubules and associates with F-Actin filaments through the intervention of motor proteins [124]. For example, the dynamic nature of mitochondria relies on the balance of fission and fusion, two processes at the basis of mitochondria remodeling that, at the biochemical level, are regulated by the activity of cytoskeletal related proteins such as Dynamin-1 like protein (Drp1), which uses its GTPase activity to achieve mitochondrial tubules constriction and dynamin 2 that finalize the fission process [91]. Other studies have correlated mechanical stress and mitochondria homeostasis in cardiac systems, and it was suggested that mitochondria have a role in mechanosensing and regulation of calcium homeostasis, which is fundamental to the contraction process [105,106,107,108,109,125,126,127].

Lysosomes are placed at the endpoint of three main pathways, namely endo-/phago-cytosis and autophagy and, as such, lysosomes receive a variety of biological components from which they gather information on their microenvironment and adapt their response to accomplish homeostatic control of cell functioning [128]. In fact, the protein aggregates breakdown process is primarily targeted to the autolysosomal compartment, and this is particularly relevant in neurodegenerative diseases where the autophagic system is often affected by mutations in key effectors such as autophagy-related (Atg) proteins [129,130]. Lysosomes impairment has been linked to a number of neurodegenerative disorders such as ALS, PD, AD and HD [131,132,133,134,135,136]. Moreover, Lysosomal Storage Disorders (LSD), which are caused by mutations in lysosomal hydrolytic enzymes and accompanied by lysosomal accumulation of undegraded substrates [137,138,139,140,141,142], are associated with neurological impairments and suppression of lysosomes–autophagosomes fusion or accumulation of malfunctioning autophagosomes [143,144]. This is because, at a baseline level, the autophagic system removes cellular waste, defective organelles and pathological protein aggregates. Under specific stress circumstances, cells should be able to enhance this process. However, aggregates clearance in neurodegenerative diseases is impaired, implying that in pathological conditions (e.g., when aggregates accumulation exceeds a certain threshold) this mechanism is inhibited at some level. It is widely accepted that various cellular stresses can cause autophagy [92] and, in this regard, the role of the Hippo pathway has been studied as a key mediator between mechanical stimuli and autophagy response (Table 1).

Recent works proposed a new molecular key player that could link aggregates accumulation and autophagic defects: Bcl-2-associated athanogene 3 (BAG3) has been shown to regulate selective macroautophagy for aggregated protein clearance and, by this, govern cellular protein quality control [111]. Several studies demonstrated that BAG3 overexpression is correlated with mutant aggregation-prone proteins such as mutant SOD1 in ALS, mutant Huntingtin in HD, and Tau in AD, suggesting that intracellular stress caused by aggregates is directly implicated in lysosomal functioning and homeostasis [112,113,114,115].

Cell mechanics control not only autophagy but also the endocytic system, which is closely related to lysosome homeostasis. In fact, the dysfunction of the endo-lysosomal system is known to be related to LSD and neurodegenerative disorders pathogenesis [118,145]. As the process of endo/exocytosis and lysosome secretion entails the modification and remodeling of the cellular membrane, it is not surprising that it is affected by mechanical alteration of stiffness, tension, physical characteristics of the cargo (e.g., size, shape, density) and the extracellular environment (Table 1). Furthermore, with this mechanism, cells are able to respond to mechanical stimuli and, in this way, overcome significant perturbations of their mechano-homeostasis [146].

Overall, the studies presented in this section support the importance of mechanosensing and mechanotransduction pathways in maintaining cellular organelle homeostasis, especially in the context of brain development and neurodegeneration.

## 4. Mechanosensing and Mechanotransduction in Neurodegenerative Diseases

The role of forces in driving neuronal development, axonal growth and injury repair has been extensively studied over the years [37,147,148,149,150,151,152,153]. The distinctiveness of the Central Nervous System (CNS) is that it is formed by a peculiar combination of different types of cells and ECM whose mechanical behavior is essential for its biological activity; for example, white and grey matter have different stiffness as a result of cell type inherent stiffness variability (myelinated axons and cellular bodies, respectively) [37,149]. The biomechanical properties of the brain have been studied using different approaches, such as Atomic Force Microscopy (AFM), which allows measuring forces at a nanoscale level with a simultaneous mapping of tissue mechanics at high resolution. Importantly, AFM could offer the possibility of describing physiological conditions and discriminating pathological variations in neurodegenerative diseases [154]. Tissue engineering approaches have been used to elucidate neural development and brain mechanical characteristics [155,156,157,158], together with machine learning methods that are able to build constitutive artificial neural networks (CANNs) that can account for viscoelastic effects of the brain [159] or predict mechanical brain response to traumatic injury [160,161].

Alterations in the neural microenvironment are associated with disease development, but it is unclear how these changes in cellular, biophysical and biochemical components contribute to either healing or ongoing degeneration. Studies using magnetic resonance elastography (MRE) that can be used for in vivo measurement of brain tissue viscoelasticity have revealed stiffness differences in the aging brain; analyzing the brains of adults ranging from 20 to 89, three independent studies observed a stiffness reduction of 15 Pa [162], 11 Pa [163] and 8 Pa [164] per year (with differences for different brain regions), reinforcing the concept that structural changes occur in the healthy brain during aging and that these are associated with physiological loss of cognitive functions due to tissue deterioration [165,166,167,168]. These premises allow us to understand that, in neurodegenerative illnesses, the alteration of tissues’ biomechanics plays a crucial role and is becoming increasingly important for the clarification of the pathophysiology of these diseases [168,169]. Nevertheless, whereas brain mechanical alterations correlate with aging and pathological states, it is unknown to what degree they influence the pathology. New findings in the role of mechanosensing channels such as Piezo1 and the Hippo pathway opened the road for the investigation of mechanobiology alteration in peripheral tissues that could help clarify pathological events happening in neurodegenerative diseases [58,170]. For example, the maintenance of the nervous system’s functionality relies on the correct organization of cytoskeletal proteins; neurons are constantly subjected to many different biological, chemical and physical factors that can cause cytoskeletal proteins’ instability, to the extent that some hereditary neurodegenerative diseases are linked to mutations in cytoskeletal genes that can be regarded as a main cause of the diseases. Furthermore, mutations in different genes and alteration of proteins involved in mechanosensing and mechanotransduction lead to cytoskeleton instability which contributes to the disease’s pathogenesis (Table 2) [171].

Various genetic disorders caused by mutations in genes involved in mechanosensing/mechanotransduction present neurological abnormalities, supporting their role in neurodegeneration. For example, Filamin A mutations cause a range of diseases that translate into different pathological phenotypes, including cognitive disability, neural migration abnormalities, and, more in general, neurological symptoms [203,204]; Myosin VA mutations are correlated to Griscelli Syndrome Type 1, an autosomal recessive disease that, among other symptoms, encompasses a series of neurological deficits [205,206]; genetic variants of Actin β or Actin γ genes cause the Baraitser–Winter syndrome, which is associated to neuronal migration impairment, brain malformations and developmental delay [207,208].

In the following sections, we will look at the current understanding and recent studies regarding brain and peripheral mechanotransduction and mechanosensing dysfunction in some major neurodegenerative diseases, such as AD, HD, ALS and PD, with the aim of understanding how these mechanisms influence the diseases’ development.

## 5. Mechanosensing and Mechanotransduction Pathways in Alzheimer’s Disease

AD is a non-curable neurodegenerative disease with the majority of cases (85–90%) being sporadic and just 10–15% familial and caused by mutations in key genes such as Amyloid Precursor Protein (APP), Presenilin 1 (PSEN1), and Presenilin 2 (PSEN2), involved in the metabolic pathway of APP. Even though the key hallmarks of the disease are the extracellular accumulation of β-Amyloid peptides (Aβ-42) and intracellular accumulation of hyperphosphorylated Tau neurofibrillary tangles, evidence showed that AD pathogenesis has a significant molecular complexity that also affects peripheral tissues and suggests that there are key molecular events that still need to be elucidated [209]. Among these, mechanobiology alterations have recently been described at different levels in AD, thus raising the interest in these pathways as potential new targets for AD research.

**Figure 3:**
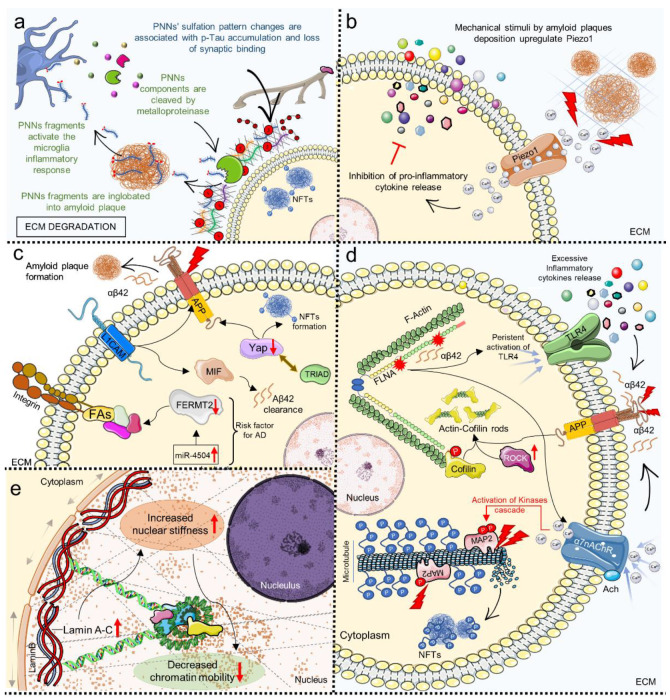
Biochemical pathways of cellular mechanosensing and mechanotransduction involved in Alzheimer’s Disease pathological mechanisms related to (**a**) The extracellular matrix (ECM); (**b**) Mechanosensitive Piezo channels; (**c**) Cellular adhesion and YAP signaling; (**d**) Cytoskeletal architecture; and (**e**) Nucleoskeletal organization (see main text for details). Red arrows up ↑ or down ↓ indicate up or downregulation. ACh, Acetylcholine; AD, Alzheimer’s Disease; APP, Amyloid Precursor Protein; Aβ42, Amyloid Beta; Ca^2+^, Calcium ion; ECM, extracellular matrix; FAs, focal adhesions; FERMT2, Fermitin family homolog 2; FLNA, Filamin A; MAP2, Microtubule-associated protein 2; MIF, macrophage migration inhibitory factor; NFTs, neurofibrillary tangles; PNNs, perineuronal nets; ROCK, Rho-associated protein kinase; TLR4, Toll-like receptor 4; YAP, Yes-associated protein 1; α7nAChR, α7 nicotinic acetylcholine receptor.

MRE analysis of AD patients’ brains revealed viscoelasticity abnormalities compared to healthy controls, such as decreased hippocampal stiffness and viscosity [210] or more, in general, softening of specific regions involved in the progression of the disease [211]. These changes might be associated with molecular mechanisms that cause ECM degradation and disruption of cytoskeletal architecture (amyloid deposition and neurofibrillary tangles formation) and, more significantly, could be used as a novel diagnostic tool [166]. Studies have shown that brain ECM composition is altered in AD (Figure 3a). For example, an important role has been given to perineuronal nets (PNNs), specialized structures containing important structural elements such as chondroitin sulfate proteoglycan (CSPG), Tenascin-R, Hyaluronic Acid, Laminin and Elastin that surround neurons and are fundamental for synaptic stability [212,213,214]. Different studies described the alteration of these structures and observed that PNNs deteriorate following diverse pathological phenomena such as AD-related microglia chronic inflammation state; the release of metalloproteinase causes the cleavage of PNNs’ components and therefore leaves neurons without their protective scaffold and more susceptible to neurotoxic agents such as Aβ plaques (Figure 3a) [214]. Furthermore, changes in the sulfation pattern of PNNs’ chondroitin sulfate-glycosaminoglycan (CS-GAG) have been linked to p-Tau accumulation and pathology progression in AD patients, suggesting that this could be an early hallmark of the disease (Figure 3a) [213].

Neuroglia and microglia cells are influenced by the stiffening of the surrounding environment caused by amyloid plaque deposition [215,216,217]. For example, studies have proven that astrocytes respond to the mechanical stimuli of amyloid plaques deposition by upregulating the mechanosensitive channel Piezo1, which seemed to cause the release of intracellular Ca^2+^ ions and the inhibition of pro-inflammatory cytokine release in AD rat models (TgF344-AD) (Figure 3b) [199,200]. Activation of Piezo1 has also been linked to CNS demyelination, while its inhibition with GsMTx4 hampered this phenomenon [57]. Although the role of up/downregulation of Piezo1 in neurodegenerative diseases still has to be fully elucidated, data suggest that this channel could be a new important therapeutic target for AD and other neurodegenerative diseases [216,217,218]. Other evidence of the effect of mechanical dysregulation in AD comes from the analysis of the Hippo pathway and, in particular, from the activation of its effector YAP [216]. Recent studies highlighted that expression of Hippo pathways components is downregulated in post-mortem human AD brains [219], and YAP downregulation is thought to occur at the early stages of AD, contributing to amyloid plaques formation and Tau hyperphosphorylation, suggesting an upstream regulator role in the AD molecular pathogenic pathways (Figure 3c) [217]. In a recent study, YAP downregulation has been correlated to TEAD/YAP-transcription-dependent-necrosis (TRIAD) in neurons of AD mice models after sequestration into intracellular amyloid aggregates, providing new insight into the early molecular processes that occur in AD (Figure 3c) [219,220].

Cellular mechanical homeostasis is impaired at several levels in AD. FAs have been implicated in AD’s etiology. In particular, genome-wide association study (GWAS) approaches revealed that Fermitin family homolog 2 (FERMT2 or Kindlin-2) is a genetic AD risk factor [183]. FERMT2 is required for FAs assembly and is involved in ECM adhesion, Actin stabilization, and integrin-mediated signaling, and recently has been demonstrated to interact with APP and participate in the regulation of axonal growth and synaptic function [184]. The FERMT2 allele linked with increased AD risk is downregulated by miRNAs overexpressed in AD brains (such as miR-4504), and this could affect AD pathogenesis (Figure 3c) [184].

According to multiple studies, cell adhesion molecules (CAMs) expression is altered in AD, and this might be related to pathogenic processes such as neuroinflammation and amyloid metabolism [221,222,223]. CAMs correct expression is fundamental for mediating cells’ interaction with the surrounding microenvironment. Their activation is critical for healthy intracellular signaling, and their precise control can decide the emergence of pathogenic conditions [224]. A recent study highlighted the importance of the Neural cell adhesion molecule L1 (L1CAM or CD171) in AD brains; L1CAM has been linked with several key mechanisms in neural homeostasis, including axonal growth, neuronal migration and differentiation, and has been shown to have a protecting function in AD by binding to Aβ and favoring its clearance, most likely by inducing the expression of the macrophage migration inhibitory factor (MIF) (Figure 3c) [196]. It is important to consider that the cytoskeletal structure completely deteriorates as the pathology progresses. The formation of NFTs caused by tau hyperphosphorylation is linked to microtubule disassembly leading to loss of cytoskeletal integrity which promotes their clustering, thus worsening the cells’ pathological phenotype (Figure 3d) [190]. Furthermore, other proteins associated with microtubules have been associated with cytoskeletal disruption, such as microtubule-associated protein 2 (MAP2), which can be abnormally phosphorylated and mediate neural cell death in AD (Figure 3d) [190].

Another aspect proving the importance of mechanobiology alterations in AD pathogenesis is the encouraging results from the Phase 1-2a-2b trials of Simufilam (PTI-125), a small molecule targeting an altered form of Filamin A found in the AD brain [225,226]. Filamin A (FLNA) is a scaffold protein that binds to a variety of partners (including receptors and channels) and is required for Actin filament cross-linking, making it an important participant in cellular stiffness regulation and stress response [39]. It has been discovered that a particular altered conformation of FLNA, deduced by its isoelectric focusing point (pI) shifting in transgenic mice AD models and post-mortem human AD brain, is correlated to two major pathogenic pathways in AD mediated by its association with soluble Aβ42: (1) α7 nicotinic acetylcholine receptor (α7nAChR) activation, which causes the activation of kinases cascade resulting in tau hyperphosphorylation and NFTs formations (Figure 3d); and (2) persistent activation of toll-like-receptor 4 (TLR4) that results in excessive release of proinflammatory cytokines, thus contributing to neuroinflammation (Figure 3d) [181,227]. Of note, in November 2021, two Phase 3 trials were started with the aim of testing whether targeting altered FLNA and inhibiting its binding with Aβ could improve cognitive performance in AD patients (NCT04994483 and NCT05026177) [228,229].

As previously mentioned, cytoskeletal architecture loss is a crucial factor in AD pathogenesis. Numerous cellular functions rely on the proper organization of microtubules, intermediate filaments and microfilaments. The latter is defined by Actin dynamics and the organization of its globular form (G-Actin) in filaments (F-Actin). The continuous transition between these two forms is a process spatially and temporally regulated by several stimuli, and appropriate oscillation of polymerization and depolymerization events regulates key cellular processes such as migration, cellular division and intracellular trafficking. Among the proteins that regulate this event, Cofilin is an Actin binding protein that mediates (in its dephosphorylated state) Actin disassembly, thereby increasing intracellular G-Actin available for the formation of new filaments and promoting Actin filament turnover, and has been shown to play an important role in neural development and neurodegeneration [230,231]. Studies have elucidated the role of Cofilin in AD. For example, Rush et al. observed an increase in Cofilin phosphorylation in mice models and AD patients, which led to synaptic plasticity loss and synaptotoxicity by inducing abnormal Actin stabilization, demonstrating that this could be a result of Rho-associated protein kinase (ROCK) pathway activation in concert with Aβ cellular exposure (Figure 3d) [174]. In recent years, many research groups have studied the relationship between AD hallmarks and aberrant Cofilin activation/deactivation, and while some contradictory results have been presented over the years, it is clear today that Cofilin regulation and Cofilin–Actin rods formation are key molecular events occurring in AD at a cellular level [175,176,177]. Notably, cytoskeletal disruption can be mediated by the accumulation of the prion protein observed in AD brains and by the prion-like propagation of Aβ [232,233,234,235].

Dysfunction of cell mechanics also extends to the nucleoskeleton. It is widely recognized that nucleoskeletal abnormalities and abnormal laminar protein expression are linked to the development of neurodegenerative diseases such as AD [236]. The nuclear structure is defined by an array of intermediate filament proteins known as lamins (Lamin A, C, B1, and B2), which are expressed differently in each cell type and serve as a scaffold for chromatin stabilization and thus are tightly linked to gene expression regulation, as well as giving the nucleus shape and rigidity [237,238]. Many studies have investigated nucleoskeletal changes occurring in AD that could be contributing factors to the development of the disease or a result of pathogenic processes; either way, the involvement of lamins dysregulation is now considered a well-defined disease hallmark that could serve as an early biomarker (Figure 3e) [187,188,239,240,241,242,243]. In AD brains, for example, Lamin A expression is increased, resulting in greater nuclear stiffness and decreased chromatin mobility (Figure 3e) [187]. This has been proposed to have a pivotal role in cell cycle re-entry [240], a phenomenon observed in AD that was previously thought to be a deleterious event associated with neuronal death but has recently been proposed to have a protective function against Aβ toxicity [244,245].

## 6. Mechanosensing and Mechanotransduction Pathways in Huntington’s Disease

**Figure 4:**
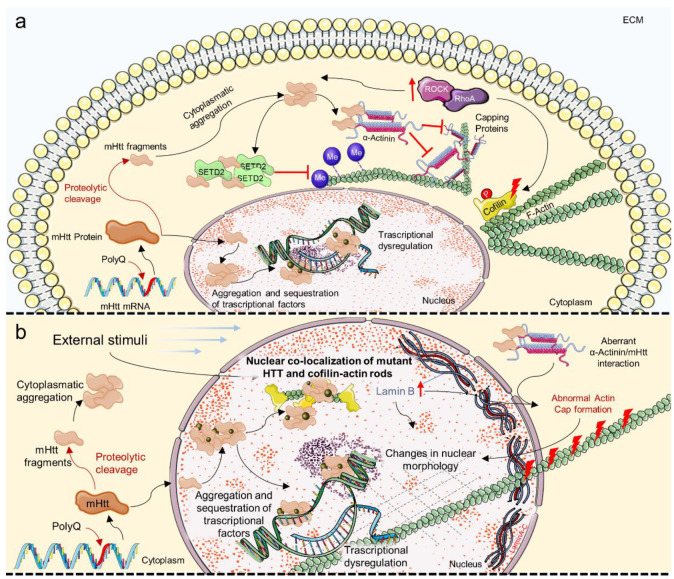
Biochemical pathways of cellular mechanosensing and mechanotransduction involved in Huntington’s Disease pathological mechanisms: (**a**) Molecular mechanism of mutant Huntingtin (mHTT) aggregates formation and their effect on cytoskeletal homeostasis and Actin dynamic, and on (**b**) nuclear architecture and functioning (see main text for details). Red arrows up ↑ indicate upregulation. Me, Methyl; mHTT, mutant Huntingtin; PolyQ, Polyglutamine; RHOA, Ras homolog family member A; ROCK, Rho-associated protein kinase; SETD2, Histone-lysine N-methyltransferase SETD2.

HD is a neurodegenerative disorder caused by mutations in the Huntingtin (HTT) gene; an aberrant expansion of the Cytosine/Adenine/Guanine (CAG) triplet in exon 1 produces HTT proteins with polyglutamine domains which become pathological when exceeding 35 repeats. HTT mutation is dominantly inherited and has a different seriousness depending on the number of repeats; low penetrance, moderate phenotype, and late-onset are associated with 36–39 repetitions, complete penetrance and late-onset are associated with 40 repeats, juvenile-onset is associated with 60 repeats, and pediatric-onset is associated with >80 repeats [246]. HD manifests with the progressive development of choreiform involuntary movements, dystonia, dementia, psychiatric disorders and brain atrophy, which lead to death 10–30 years after the diagnosis, depending on the severity of the disease. HTT is expressed ubiquitously in the human body, but its higher expression is in the brain. Despite the lack of a specific definition of HTT function, it has been demonstrated that HTT is involved in CNS development, synaptic activity, axonal trafficking and cell survival, and hence its mutation is associated with neuronal death [247,248]. Because HTT appears to have a scaffolding role for many proteins, a putative role of HTT might be to coordinate many biological activities. As a result, its mutation disrupts physiological protein–protein interactions causing HTT to accumulate intracellularly and affecting essential biological processes (Figure 4a,b) [248,249].

Cofilin–Actin rods formation has been reported in HD. The observation of nuclear co-localization of mutant HTT and Cofilin–Actin rods in mice indicated that mutant HTT could play a role in cytoskeletal reorganization after external stimuli such as heat or stress (Figure 4b) [177]. Actin stabilization and remodeling appear to be crucial in HD. The sustained activation of the RhoA/ROCK signaling pathway has been linked to the formation of HTT aggregates and the pathological regulation of its downstream cytoskeletal proteins, such as Cofilin (Figure 4a) [178]. HTT intracellular accumulation has been demonstrated to disrupt epigenetic pathways, particularly those involving Actin methylation. Actin polymerization and cell migration have been shown to be dependent on correct Actin methylation by Histone-lysine N-methyltransferase SETD2 (SETD2), which only occurs when SETD2 interacts with HTT; mutant HTT appears to sequester SETD2 and inhibit its function, exacerbating pathological mechanisms by interfering with key cellular mechanisms like chromatin regulation and cell migration [182]. In another recent work, Actin dynamic perturbation has been linked to HD. Primary fibroblasts from HD patients have been shown to have a unique nuclear morphology that is linked to a lack of nuclear Actin caps, which inhibit cell motility (Figure 4a,b) [172]. In particular, abnormal Actin cap formation could be due to aberrant interaction between mutant HTT and α-Actinins, a component of the perinuclear Actin cap [250]. In this model, HTT controls the distribution of α-Actinins and integrates molecular growth pathways to Actin assembly and adhesion dynamics (Figure 4a,b) [172]. Lamin B, engaged directly in Actin cap formation as anchoring for F-Actin, plays a crucial role in HD nuclear homeostasis; neurons from the HD mouse model showed an increase in Lamin B1 expression, which correlated with nuclear morphology and transport alterations that were restored after Lamin B1 normalization (Figure 4b) [188].

## 7. Mechanosensing and Mechanotransduction Pathways in Amyotrophic Lateral Sclerosis

**Figure 5:**
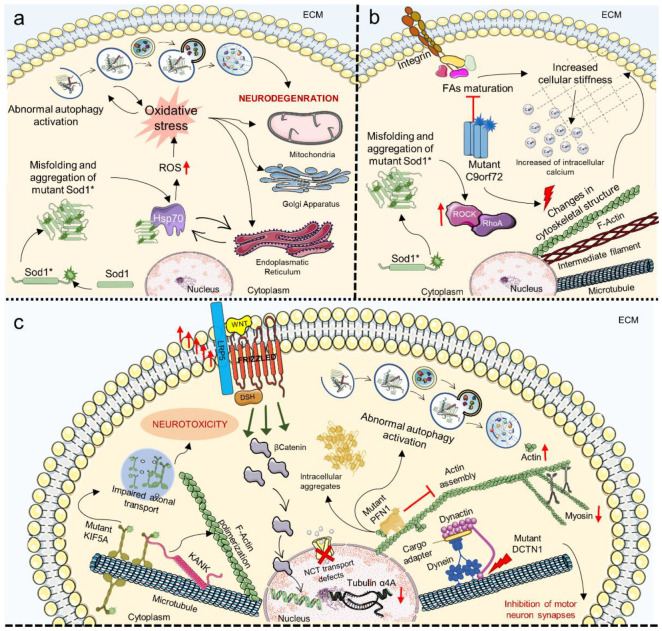
Biochemical pathways of cellular mechanosensing and mechanotransduction involved in Amyotrophic Lateral Sclerosis (ALS) pathological mechanisms: (**a**) Pathogenic molecular mechanism of mutant (*) Superoxide Dismutase 1 (Sod1*) and effects on cellular homeostasis; (**b**) Effect of Sod1* and mutant C9orf72 dipeptides aggregation on cellular adhesion, RhoA/ROCK signaling and cytoskeletal structure; (**c**) Biochemical mechanotransduction pathways affecting cytoskeletal and nuclear homeostasis evidenced in ALS (see main text for details). Red arrows up ↑ or down ↓ indicate up or downregulation. C9Oorf72, Chromosome 9 open reading frame 72; Ca^2+^, Calcium ion; DCTN1, Dynactin Subunit 1; DSH, Dishevelled; FAs, focal adhesions; HSP70, Heat shock protein 70; KANK, KN motif and ankyrin repeat domain-containing protein 1; KIF5A, cytoskeleton-related proteins such as Kinesin; LRP5, LDL Receptor Related Protein; NCT, nucleocytoplasmic; PFN1, Profilin 1; SOD1*, mutant Superoxide Dismutase 1; WNT, Wingless/Integrated.

ALS is a neurodegenerative disease that usually has an adulthood onset and a rapid progression, with 90% of cases considered sporadic while up to 10% are familial (fALS) and caused by mutations in mutations in genes with a wide range of functions [251,252]. It is characterized by loss of both upper and lower motor neurons due to degeneration of the motor cortex; when neural signaling is impaired, muscle weakening leads to progressive paralysis and results in death from respiratory failure around 3 to 5 years after symptoms manifest. To date, there is not a resolutive therapeutic approach for ALS because its etiology and pathogenic molecular mechanism are still unclear. Despite the fact that various studies have identified pathways in neurons and systemic districts that are implicated in ALS throughout the years, it is still unclear which pathways are a result of the disease and which are a cause, but it is obvious that they impact a range of motor neuron activities [251,252,253]. Among the genes involved in fALS, the most common are mutant superoxide dismutase 1 (SOD1) chromosome 9 open reading frame 72 (C9orf72), TAR DNA binding proteins (TDP43), and fused in sarcoma (FUS). In particular, mutant SOD1, TDP-43, and FUS proteins have a strong propensity to form aggregates that consist of intracellular inclusions with a tendency to form prion-like propagating fibrils, which disrupt cell homeostasis on multiple levels [253,254]. It is known that aggregation of these protein cause oxidative stress, which affects cellular organelles such as the ER, Golgi and mitochondria by interfering with their homeostatic metabolic functions. SOD1 accumulation induces reactive oxidative species (ROS) production via diverse mechanisms such as sequestration of key chaperones (i.e., Hsp70) and alteration of membranes interaction, leading to disruption of cell bioenergetics and abnormal autophagy activation resulting in neurodegeneration [255,256,257,258]; as the ER is involved in protein biosynthesis and many chaperones are present in this organelle, its malfunctioning is directly linked to the propagation of protein aggregation and misfolding (Figure 5a) [254]. Studies regarding Tar DNA-binding protein 43 (TDP-43), which accumulates in certain neurodegenerative diseases such as ALS and Frontotemporal dementia (FTD), showed that it was found in mitochondria where it led to aberrant morphology and mitophagy mediated by Parkin, increased production of ROS, mitochondrial membrane permeabilization and impaired oxidative phosphorylation [195,259]. It was proven that the C-Terminal fragments (TDP-25) co-localized with Myosin IIb in mitochondria and that Myosin IIb inhibition resulted in the buildup of insoluble TDP-25 in the mitochondria and decreased the survival of neuronal cells, which could be a possible therapeutic target [195]. Of note, the RhoA/ROCK signaling pathway has been shown to be overactive in animal models and patients with ALS, contributing to motor neurons’ death (Figure 5b) [178].

As chaperone assisted proteostasis is critical for mechanosensing and mechanotransduction mechanisms, protein aggregation is a key component in the study of the relationship between ALS and changes in cell mechanics [260]. For example, a recent study correlated dipeptides produced by pathogenic C9orf72 mutation with changes in cytoskeletal architecture and FAs maturation, which led to increased cellular stiffness by increasing intracellular calcium concentration (Figure 5b) [260]. The involvement of the cytoskeleton in the development of ALS has been supported by GWAS identification of mutations on cytoskeleton-related proteins such as Kinesin 5A (KIF5A), required for axonal transport, and the Actin polymerization regulating protein KN motif and ankyrin repeat domain-containing protein 1 (KANK1), which were shown to correlate with disease severity (Figure 5c) [185,186]. The preservation of correct synaptic functioning depends on the dynamic cytoskeletal regulation of molecules and organelles’ movement through the axon, which is fundamental for the motor neurons’ homeostasis. In this panorama, other genes linked to both familial and sporadic ALS affecting cytoskeletal dynamics are: (i) Alsin Rho Guanine Nucleotide Exchange Factor (ALS2), involved in the disruption of endosomal dynamics and induction of neurite outgrowth [261]; (ii) DynActin Subunit 1 (DCTN1), which interacts with dynein and microtubules to regulate intracellular transport and when mutated hinders synapsis formation of motor neurons (Figure 5c) [262]; (iii) Profilin 1 (PFN1) that promotes Actin polymerization and when mutated interferes with the autophagic pathway and forms intracellular aggregates (Figure 5c) [263]; (iv) intermediate filament genes such as neurofilament light and heavy chains, mutated in patients with ALS [264]; and (v) Tubulin Alpha 4A that was found to be less transcribed in the brain and spinal cord of patients with both familial and sporadic ALS (Figure 5c) [173,265,266].

Tubulin acetylation is involved in the pathogenic mechanisms of ALS since its dysregulation perpetuates SOD1 aggregation and hinders axonal transport [267]. The importance of cytoskeletal architecture also passes by the maintenance of proper actomyosin complexes, which are especially important in ALS due to their function in the motor system; it has been shown that primary myotubes from transgenic ALS mice have different mechanical characteristics compared to WT with an increase in elastic modulus values that appear to occur early in the disease pathogenesis, with an increase in Actin and a decrease in Myosin gene expression (Figure 5c) [194].

Actin dynamic homeostasis has been associated with nucleocytoplasmic transport defects and correlated with motor neurons dysfunction in ALS caused by mutations in PFN1 and C9orf72 (Figure 5c) [268]. Modulation of Actin polymerization disrupts the nuclear pore complex (NPC), resulting in neuronal degeneration. This phenomenon might be particularly important to the disease’s etiology, bringing up novel treatment targets for various forms of ALS [269]. Indeed, malfunctioning of NPC mediated transport was observed in primary and iPSCs-derived neurons with mutant or aggregated TDP-43 protein where nucleoporins and transport factors are sequestered, thus impairing RNAs import and export and directly linking protein accumulation and nucleocytoplasmic transport [197,198,270]. Mutations in ALS may alter the nuclear structure at different levels, such as the nuclear lamina, Nups and NPC, Ran and its regulators, all resulting in defective nucleocytoplasmic transport (Figure 5c) [271].

Finally, among the soluble intermediates involved in mechanical stimuli transduction, the WNT pathway, which among other functions is important in mediating cellular response to external loading and strain, has been shown to be hyperactivated in the spinal cord of the SOD1^G93A^ mouse model, though the effect of this is not yet clear (Figure 5c) [74,267].

## 8. Mechanosensing and Mechanotransduction Pathways in Parkinson’s Disease

**Figure 6:**
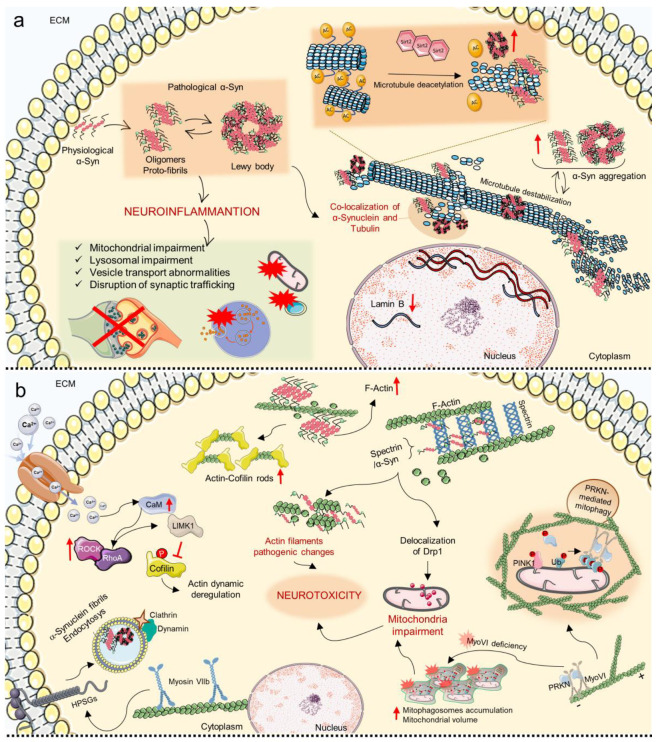
Biochemical pathways of cellular mechanosensing and mechanotransduction involved in Parkinson’s Disease pathological mechanisms: (**a**) Pathogenic molecular mechanism of α-Synuclein aggregation and Lewy bodies formation and effects on cellular homeostasis, microtubules dynamic, nucleoskeletal architecture, and on (**b**) cytoskeletal Actin dynamic and its involvement in mitochondria homeostasis and α-Synuclein endocytosis (see main text for details). Red arrows up ↑ or down ↓ indicate up or downregulation. AC, Acetyl; Ca^2+^, Calcium ion; CAM, cell adhesion molecules; DRP1, Dynamin-1 like protein; HSPGs, Heparan sulfate proteoglycans; LIMK1, LIM Domain Kinase 1; MyoVI, Myosin VI; Parkin RBR E3 Ubiquitin Protein Ligase; PIN K, PTEN Induced Kinase 1; RHOA, Ras homolog family member A; ROCK, Rho-associated protein kinase; SIRT2, Sirtuin 2; UB, Ubiquitin; PRKN, Parkin RBR E3 Ubiquitin Protein Ligase; α-Syn, α-Synuclein.

PD is one of the most prevalent neurodegenerative diseases, second only to AD, the incidence of which has been rapidly increasing over the last decade [272,273]. The main symptoms of PD are motor-related (e.g., tremor and bradykinesia), accompanied by non-motor symptoms such as depression, and have an onset that increases with age (5–10% before 50 years old, 25% under 65 years old). The etiology of the disease is highly complex, encompassing abnormal accumulation of the α-Synuclein protein, mitochondrial, lysosomal, and vesicle transport abnormalities, disruption of synaptic trafficking, and neuroinflammation, all leading to the death of dopaminergic neurons, especially in the substantia nigra (Figure 6a). Only 10–15% of PD cases have a genetic cause, with the principal mutations involving monogenic causes such as Synuclein Alpha (SNCA), VPS35 Retromer Complex Component (VPS35), PTEN Induced Kinase 1 (PINK1), Parkinsonism Associated Deglycase (PARK7), Parkin RBR E3 Ubiquitin Protein Ligase (PRKN) and Leucine Rich Repeat Kinase 2 (LRRK2) and other genetic variants that contribute to the development of the pathology [274]; SCNA, PRKN and LRRK2 are linked to microtubule destabilization, and in different studies, the co-localization of α-Synuclein and Tubulin has been observed in pathological inclusions of α-Synuclein such as Lewy bodies [275,276,277]. In this regard, α-Synuclein/Tubulin interaction has been discussed for several years now, but the mechanisms underlying are still not completely understood; most likely, microtubule alterations influence the development and storage of α-Synuclein aggregates, whereas, at the same time, α-Synuclein overexpression causes cytoskeletal microtubule impairments and neurodegeneration (Figure 6a) [277].

In a recent study, the toxin 2,5-hexanedione (2,5-HD) was used to induce neurodegeneration in fibroblasts from PD donors with mutations in the PRKN gene and in mice models, and alterations in the stability and architecture of microtubules were observed [278]. In particular, microtubules were shown to accumulate as a consequence of network fragmentation and to have a different pattern of post-translational modification (detyrosination and acetylation), an index of impaired tubulin dynamic [278].

Tubulin acetylation has been recognized as a central regulator of cellular homeostasis in neurodegenerative diseases as in vitro and in vivo PD models’ axonal transport was demonstrated to be restored by increasing microtubule acetylation. Studies using Sirtuin 2 (a tubulin deacetylase) inhibitors confirmed that induction of microtubule acetylation has a protective role from α-Synuclein toxicity, reducing astrocytes’ reactivity and favoring dopaminergic neurons’ survival (Figure 6a) [202,275]. Together with Tubulin, other cytoskeletal components have been proven to have a central role in PD pathogenesis. For example, Spectrin binds α-Synuclein leading to Actin dynamic impairments and mitochondria loss of function through delocalization of Drp1; in mice and post-mortem human brains with α-Synucleinopathy, it was shown that Spectrin overexpression rescues α-Synuclein toxicity and restores cytoskeletal architecture homeostasis, demonstrating that α-Synuclein/Spectrin association causes Actin filaments’ pathogenic changes and leads to consequent neurotoxicity, and offering a new potential therapeutic target (Figure 6b) [201]. Additionally, transgenic flies with α-Synuclein accumulation showed the presence of Actin–Cofilin rods and increased Actin filaments in brains (Figure 6b) [201]. These rod-like structures were seen in some particular brain regions of PD animal models harboring α-Synuclein mutations and in patients with Lewy–Bodies dementia, confirming the fundamental role of cytoskeletal correct Actin architecture and Cofilin activity for maintaining neural correct functioning [179]. Moreover, mutations in the DCTN1 gene cause a particular form of PD called Perry Syndrome, a disease characterized by levodopa resistance, weight reduction, mental confusion and central respiratory distress, in which DCTN1 loses its affinity to microtubules and results in impaired neuronal transport [180].

The role of cytoskeletal homeostasis in PD pathogenesis is not limited to Actin’s organization but also relates to other important cellular architectural components. For instance, a recent study indicated a relationship between Myosin VI and impaired mitochondria activity, which is particularly significant considering that mitochondria are extremely dynamic structures that are associated with several cellular activities and that mitochondrial failure has gained an increasingly important role in the pathogenesis of neurodegenerative disorders [123,279]. Mitochondrial homeostasis is fundamental for preserving cellular homeostasis, which is accomplished by eliminating ubiquitinated, damaged mitochondria by PRKN-mediated mitophagy. Myosin VI associates with the negative end of Actin filaments, binds to PRKN to create a complex, and is then specifically drawn to defective mitochondria through its ubiquitin-binding domain; in order to surround these mitochondria with diminished respiratory capacity and oxidative phosphorylation potential, Myosin VI triggers the formation of F-Actin enclosures that create a physical barrier with the rest of the cytoplasmatic content and inhibit their fusion with healthy mitochondria (Figure 6b) [279,280,281]. Of note, Myosin VI deficiency causes mitophagosomes accumulation and mitochondrial volume increase (Figure 6b) [279]. Mitochondrial dysfunction has been observed in α-Synucleinopathy animal models and post-mortem human brains consequent to a-Synuclein accumulation and Actin reorganization through Spectrin and altered Drp1 localization; Drp1 overexpression in animal models was sufficient to rescue motor deficit and mitochondrial homeostasis (Figure 6b) [201].

In another study, Myosin-VIIB was proposed to be fundamental in the endocytosis of α-Synuclein fibrils. It was shown that Myosin-VIIB binds Actin and stimulates its assembly and stabilization within membranes’ heparan sulfate proteoglycans (HSPGs), allowing the formation of protrusions that ultimately surround cargos forming clathrin-coated pits; with the intervention of dynamin, these protrusions are cut and vesicles are released inside the cytoplasm mediating the entrance of α-Synuclein fibrils fibers (Figure 6b) [282].

Mechanobiology alterations that contribute to the pathogenesis of PD have been described at the nuclear level. Considering the established assumption that senescent cells exhibit nuclear lamina alterations which lead to morphological and gene expression changes [283,284], studies investigated laminar dysfunctions in PD and suggested a correlation with its pathophysiology. Reduction of Lamin B1 was observed in PD astrocytes of post-mortem human brain and toxin-treated mice but not in other surrounding cell types, suggesting that senescence may be a contributing factor to PD (Figure 6a) [189,285].

Regarding soluble mechanical transducers involved in the pathogenesis of PD, several works demonstrated that activation of the RhoA signaling pathway has an important role in dopaminergic loss and axonal disruption in PD [178].

In the presence of an increased intracellular Calcium influx, the calcium-calmodulin axis activates and starts a phosphorylation cascade that leads to activation of RhoA and its effector ROCK that in turn activates the LIM Domain Kinase 1 (LIMK1), a Cofilin inhibitor; when Cofilin activity is impaired, neurons undergo Actin dynamic deregulation and loss of cytoskeletal architecture, which may contribute to axonal degeneration and loss of dopaminergic neurons in PD (Figure 6b) [286]. Moreover, it was shown that in hIPSCs harboring mutations in PARK2, the RhoA pathway was significantly increased and was proposed to be linked to impaired migration and neuritogenesis, as its inhibition rescued this pathogenic phenotype [287]. As a matter of fact, transient receptor potential channels that are involved in ions’ transport, such as calcium, and that can be activated by a plethora of mechanical stimuli, are today considered promising targets for PD treatments [288].

## 9. Concluding Remarks

In this review, we discussed recent evidence with the intent of clarifying how mechanosensing and mechanotransduction mechanisms are involved in the pathogenesis of neurodegenerative diseases. In particular, our interest was to focus our attention on those diseases that encompass metabolic accumulation of mutated proteins and have as primary characteristics the formation of pathological intracellular aggregates. This is because the presence of intracellular inclusion is believed to affect cells’ mechanical characteristics which, in turn, deeply influence cellular biochemical signaling and, in this way, participate in the pathogenic mechanism of neurodegenerative diseases.

We presented several important works that helped elucidate how cells’ homeostasis is inextricably linked to the balance of their mechanical properties, which highlighted that the study of cellular organelles’ homeostasis and dysfunction is important to understand the mechanotransduction’s axes. Although part of the crosstalk mechanisms between soluble signaling molecules and mechanical forces has been elucidated, much more research still needs to be done to understand to what extent mechanobiology alterations can be considered the cause or result of some pathological features. One of the hurdles that make this question particularly hard to answer is the limitation of experimental methods available to study in vitro single-cell responses to internal forces’ changes. Indeed, most of the research has concentrated on mechanotransduction response related to alterations of external environments through the use of biomaterials and novel 3D culture systems. Thanks to these bio-hybrid systems and gene editing techniques, some pathways of the outside–inside molecular signaling in response to mechanical stimuli have been described. However, little is known about how cells respond to changes in internal mechanical characteristics and how these influence cellular behavior and gene expression. Notably, the clarification of these processes would be of great importance in the perspective of identifying new molecular key players that could offer novel therapeutic targets for complex neurodegenerative diseases for which there is still no effective treatment.

Together with the previously mentioned phase 3 clinical trials for AD involving the targeting of altered FLNA and its binding with Aβ [228,229], several emerging and promising therapeutic strategies for neurodegenerative diseases are targeted to the prevention or elimination of the aggregation events that occur extra- and intra-cellularly [289,290,291,292,293,294]. Nevertheless, the identification of disease-modifying agents is still not accomplished, which reinforces the necessity of a deeper comprehension of biochemical pathways of cellular mechanosensing/mechanotransduction mechanisms and their role in neurodegenerative disease pathogenesis.

## Figures and Tables

**Table 1:** Intracellular transmission of mechanosensing and mechanotransduction pathways: the involvement of organelles’ homeostasis and dysfunction.

Organelle	Mechanosensing and Mechanotransduction Pathways Involved in Organelles’ Homeostasis and Dysfunction	Ref.
Endoplasmic Reticulum (ER) 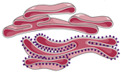	• Expression of the mechanosensitive channel PANX1 and response to ultrasound stimuli by releasing the signaling molecule Ca^2+^	[95]
	• ER localization of Piezo1 in response to ER membrane tension mediates Ca^2+^ release	[96]
Golgi Apparatus 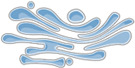	• Loss of cell adhesion causes Golgi fragmentation and loss of functioning in an integrin-mediated way through the modulation of the Arf1 activity • Increased glycosylation and trafficking of plasma membrane proteins	[97]
	• RhoA pathway activation is correlated to trans-Golgi vesicles fission via a signaling pathway involving microtubules depolymerization, Myosin IIa and GEF-H1 in integrin-mediated adhesion	[98]
	• Lipid metabolic activity modification in response to external mechanical stimuli	[99]
Mitochondria 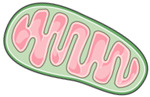	• Suspension culture of epithelial cells induces a reduced usage of glucose for the TCA cycle, which leads to lower ATP production restored by overexpression of ERBB2, an oncogene	[100]
	• Spheroids from mammary epithelial cells enhance proline metabolism in order to maintain ATP synthesis and intensify the antioxidant activity of mitochondria	[101]
	• Cell detachment in cancer cells stimulates mitophagy, a particular type of autophagy targeted to mitochondria, that was proposed to be regulated by the serine/threonine kinase 1 RIPK1, which increases ROS generation and drives non-apoptotic cell death	[100,101,102]
	• Mechanical perturbation of cells with intracellular pathogens or extracellular stimulation with AFM compression engages the fission complex via the mitochondrial fission factor mitochondrial membrane	[103]
	• Decreasing mitochondrial tension by microtubules depolymerization and Myosin II inhibition reduces the probability of mitochondrial fission	[104]
	• FAs Kindlins mitochondrial accumulation in response to ECM stiffening • F-Actin polymerization around mitochondria-ER contact point induces mitochondrial constriction and fission	[100]
	• Prolonged mechanical stress causes an increase in glycolysis and glucose oxidation in CMs leading to impairment of mitochondria functioning and compromised ETC	[105,106]
	• CMs integrins respond to excessive mechanical load with the involvement of the MAPK and RhoA pathway, which results in ETC dysfunction and insufficient ATP synthesis	[107,108,109]
	• YAP activation with Melatonin favors mitochondrial fusion	[109]
Lysosomes, Autophagy Endo-lysosomal system 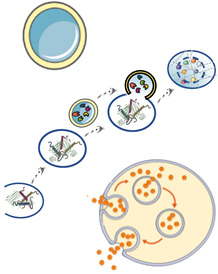	• YAP/TAZ activation simulates autophagy by inducing the expression of a RAB7 inhibiting protein, Armus, necessary for the activation of the autophagy flux	[84]
	• Contact inhibition of cells cultured at high density on soft matrices showed to induce autophagy impairment through inhibition of YAP/TAZ activity axis with consequent loss of stress fibers and MyosinII that maintain the kinases LATS1/2 active	[110]
	• Intracellular stress caused by aggregates is implicated in lysosomal functioning and autophagy defects through BAG3 expression	[111,112,113,114,115]
	• Mutations leading to misfolded FLNC induce its intracellular accumulation, leading to autophagy activation and increasing lysosomes’ expression in human cardiomyocytes	[116]
	• Soft ECM impairs autophagosomes formation	[100]
	• Membrane tension regulates the CLIC/GEEC (CG) endocytic pathway through membrane-bound Vinculin that mediates its activation (with high membrane tension) or inhibition (with low tension)	[117]
	• Defects in internalization, recycling and lysosomal degradation through the endo-lysosomal compartment of integrins are correlated to pathologic conditions such as cancer and inflammation	[118]
	• Extracellular vesicles deriving from arthritic chondrocytes transport miR-221 and act as mediators of mechanical signaling and inhibiting in vitro bone development	[119]
	• Accumulation of substrates impose a perturbation in the homeostatic rheology of the cell that causes inhibition of lysosomal trafficking; following microtubules disassembly, enlarged lysosomes with prolonged ER-contact sites are retained in the cytoplasm of fibroblasts from MPS-1 and -3B patients	[120]
	• Lysosomal trafficking is regulated by substrate stiffness via different molecular adaptors; LRRK1 induces retrograde transport and perinuclear accumulation in soft matrices while VARP mediates exocytosis in rigid substrates	[121]
	• VAMP7 vesicles are important for the regulation of the plasma membrane composition in terms of glyco- and sphingolipids, correlating the sensing of environmental mechanical characteristics and the cellular biochemical response that leads to changes in adhesion and integrin dynamic	[121]
	• LAMP1 positive extracellular vesicles release is regulated by increased calcium influx induced by excessive mechanical stress	[122]

AFM, atomic force microscopy; Arf1, ADP-ribosylation factor 1; ATP, Adenosine Triphosphate; BAG3, BCL-2-associated athanogene 3; CMs, cardiomyocytes; ECM, extracellular matrix; ERBB2, Erb-B2 Receptor Tyrosine Kinase 2; ETC, electron transport chain; FAs, focal adhesions; FLNC, Filamin C ; GEF-H1, Rho guanine nucleotide exchange factor 2; LAMP1, Lysosomal-associated membrane protein 1; LATS1/2, large tumor suppressor kinase 1 and 2; LRRK1, Leucine Rich Repeat Kinase 1; MAPK, Mitogen-activated protein kinase; MPS-1, Mucopolysaccharidosis type 1; MPS-3B, Mucopolysaccharidosis type 3B; PANX1, Pannexin 1; RAB7, Ras-related protein Rab-7; RhoA, Ras homolog family member A; RIPK1, receptor interacting serine/threonine kinase 1; TAZ, transcriptional co-activator with PDZ-binding motif; TCA, the tricar-boxylic acid cycle; VAMP7, vesicle-associated membrane protein 7; VARP, VPS9 domain and ankyrin repeat-containing protein; YAP, Yes-associated protein 1.

**Table 2:** Mechanosensing/mechanotransduction related genes and proteins altered in neurodegenerative diseases.

Genes/Proteins	Function in Homeostasis	Type of Alteration	Neurodegenerative Pathology	Ref.
α-Actinin	Scaffolding protein involved in Actin crosslinking	Protein delocalization	HD	[172]
Alsin Rho Guanine Nucleotide Exchange Factor	Guanine-nucleotide exchange factor, regulates GTPase activity	Gene mutations (loss of function)	ALS	[173]
Cofilin	When dephosphorylated mediates F-Actin disassembly	Increase/decrease in protein activity	AD	[174,175,176,177]
		Increase protein activity	HD	[178]
		Decrease in protein activity	PD	[179]
DynActin Subunit 1	Mediates vesicles retrograde transport by interacting with Dynein	Gene mutations	ALS	[173]
			Perry Syndrome	[180]
Filamin A	Scaffold protein required for F-Actin cross-linking	Altered conformation	AD	[181]
Histone-lysine N-methyltransferase SETD2	Actin methylation	Protein activity inhibition	HD	[182]
Kindlin-2	Required for FAs assembly and involved in ECM adhesion, Actin stabilization, and integrin-mediated signaling	Gene downregulation	AD	[183,184]
Kinesin 5A	Motor protein involved in spindle formation	Loss-of-function mutations	ALS	[185]
KN motif and ankyrin repeat domain-containing protein	Actin polymerization regulation	Gene mutations	ALS	[186]
Lamin A	Structural protein of the nuclear envelope	Protein upregulation	AD	[187]
Lamin B	Structural protein of the nuclear envelope	Protein upregulation	HD	[188]
		Protein downregulation	PD	[189]
Microtubule-associated protein 2	Essential for microtubule (MTs) assembly through crosslinking with intermediate filaments	Protein hyperphosphorylation	AD	[190,191]
		Splicing Alteration	HD	[191,192]
		Increased protein levels in CSF	ALS	[193]
Myosin heavy chain	Motor protein fundamental for cellular contractility	Decreased protein expression	ALS	[194]
Myosin IIb	Motor protein involved in Actin organization	Co-localization with TDP-25	ALS	[195]
Neural cell adhesion molecule L1	Axonal growth, neuronal migration and differentiation	Protein downregulation	AD	[196]
Neurofilament light and heavy chains	Neural intermediate filaments	Gene mutations	ALS	[173]
Nucleoporins	Mediate nucleocytoplasmatic transport	Protein sequestration	ALS	[197,198]
Piezo 1	Mechanosensitive ion channel, mediates Ca^2+^ cellular influx	Protein upregulation	AD	[199,200]
Profilin 1	Promotes Actin polymerization	Gene mutations	ALS	[173]
Spectrin	Structural protein of the cell membrane	Protein binding by α-Synuclein	PD	[201]
Tubulin Alpha 4A	Microtubules component	Gene mutations	ALS	[173]
Tubulin	Microtubules constituent	Protein acetylation	PD	[202]

AD, Alzheimer’s Disease; ALS, Amyotrophic Lateral Sclerosis; HD, Huntington’s Disease; PD, Parkinson’s Disease.

## Data Availability

Not applicable.

## References

[1] Niethammer P. (2021). Components and Mechanisms of Nuclear Mechanotransduction. Annu. Rev. Cell Dev. Biol..

[2] Pennacchio F.A., Nastały P., Poli A., Maiuri P. (2021). Tailoring Cellular Function: The Contribution of the Nucleus in Mechanotransduction. Front. Bioeng. Biotechnol..

[3] Motz C.T., Kabat V., Saxena T., Bellamkonda R.V., Zhu C. (2021). Neuromechanobiology: An Expanding Field Driven by the Force of Greater Focus. Adv. Healthc. Mater..

[4] Park S., Jung W.-H., Pittman M., Chen J., Chen Y. (2020). The Effects of Stiffness, Viscosity, and Geometry of Microenvironment in Homeostasis, Aging and Diseases. J. Biomech. Eng..

[5] Tortorella I., Argentati C., Emiliani C., Martino S., Morena F. (2021). The role of physical cues in the development of stem cell-derived organoids. Eur. Biophys. J..

[6] Argentati C., Tortorella I., Bazzucchi M., Morena F., Martino S. (2020). Harnessing the Potential of Stem Cells for Disease Modeling: Progress and Promises. J. Pers. Med..

[7] Abdeljawad M.B., Carette X., Argentati C., Martino S., Gonon M.-F., Odent J., Morena F., Mincheva R., Raquez J.-M. (2021). Interfacial Compatibilization into PLA/Mg Composites for Improved In Vitro Bioactivity and Stem Cell Adhesion. Molecules.

[8] Argentati C., Morena F., Fontana C., Tortorella I., Emiliani C., Latterini L., Zampini G., Martino S. (2021). Functionalized Silica Star-Shaped Nanoparticles and Human Mesenchymal Stem Cells: An In Vitro Model. Nanomaterials.

[9] Morena F., Argentati C., Soccio M., Bicchi I., Luzi F., Torre L., Munari A., Emiliani C., Gigli M., Lotti N. (2020). Unpatterned Bioactive Poly(Butylene 1,4-Cyclohexanedicarboxylate)-Based Film Fast Induced Neuronal-Like Differentiation of Human Bone Marrow-Mesenchymal Stem Cells. Int. J. Mol. Sci..

[10] Luzi F., Tortorella I., Di Michele A., Dominici F., Argentati C., Morena F., Torre L., Puglia D., Martino S. (2020). Novel Nanocomposite PLA Films with Lignin/Zinc Oxide Hybrids: Design, Characterization, Interaction with Mesenchymal Stem Cells. Nanomaterials.

[11] Argentati C., Morena F., Montanucci P., Rallini M., Basta G., Calabrese N., Calafiore R., Cordellini M., Emiliani C., Armentano I. (2018). Surface hydrophilicity of poly(l-Lactide) acid polymer film changes the human adult adipose stem cell architecture. Polymers.

[12] Morena F., Armentano I., Montanucci P., Argentati C., Fortunati E., Montesano S., Bicchi I., Pescara T., Pennoni I., Mattioli S. (2017). Design of a nanocomposite substrate inducing adult stem cell assembly and progression toward an epiblast-like or primitive endoderm-like phenotype via mechanotransduction. Biomaterials.

[13] Aragona M., Sifrim A., Malfait M., Song Y., Van Herck J., Dekoninck S., Gargouri S., Lapouge G., Swedlund B., Dubois C. (2020). Mechanisms of stretch-mediated skin expansion at single-cell resolution. Nature.

[14] Xia P., Gütl D., Zheden V., Heisenberg C.-P. (2019). Lateral Inhibition in Cell Specification Mediated by Mechanical Signals Modulating TAZ Activity. Cell.

[15] Pocaterra A., Santinon G., Romani P., Brian I., Dimitracopoulos A., Ghisleni A., Carnicer-Lombarte A., Forcato M., Braghetta P., Montagner M. (2019). F-actin dynamics regulates mammalian organ growth and cell fate maintenance. J. Hepatol..

[16] Mohammed D., Versaevel M., Bruyère C., Alaimo L., Luciano M., Vercruysse E., Procès A., Gabriele S. (2019). Innovative Tools for Mechanobiology: Unraveling Outside-In and Inside-Out Mechanotransduction. Front. Bioeng. Biotechnol..

[17] Scheuren A., Wehrle E., Flohr F., Müller R. (2017). Bone mechanobiology in mice: Toward single-cell in vivo mechanomics. Biomech. Model. Mechanobiol..

[18] Verbruggen S. (2018). Mechanobiology in Health and Disease.

[19] Yu W., Sharma S., Rao E., Rowat A.C., Gimzewski J.K., Han D., Rao J. (2021). Cancer cell mechanobiology: A new frontier for cancer research. J. Natl. Cancer Cent..

[20] Liang C., Huang M., Li T., Li L., Sussman H., Dai Y., Siemann D.W., Xie M., Tang X. (2022). Towards an integrative understanding of cancer mechanobiology: Calcium, YAP, and microRNA under biophysical forces. Soft Matter.

[21] Choudhury A.R., Gupta S., Chaturvedi P.K., Kumar N., Pandey D. (2019). Mechanobiology of Cancer Stem Cells and Their Niche. Cancer Microenviron..

[22] Armistead F., De Pablo J.G., Bloomfield-Gadêlha H., Peyman S.A., Evans S.D. (2020). Physical Biomarkers of Disease Progression: On-Chip Monitoring of Changes in Mechanobiology of Colorectal Cancer Cells. Sci. Rep..

[23] Broders-Bondon F., Ho-Bouldoires T.H.N., Sanchez M.E.F., Farge E. (2018). Mechanotransduction in tumor progression: The dark side of the force. J. Cell Biol..

[24] Tschumperlin D.J., Ligresti G., Hilscher M.B., Shah V.H. (2018). Mechanosensing and fibrosis. J. Clin. Investig..

[25] Tschumperlin D.J., Lagares D. (2020). Mechano-therapeutics: Targeting Mechanical Signaling in Fibrosis and Tumor Stroma. Pharmacol. Ther..

[26] Harn H.I., Ogawa R., Hsu C., Hughes M.W., Tang M., Chuong C. (2019). The tension biology of wound healing. Exp. Dermatol..

[27] Humphrey J.D., Schwartz M.A. (2021). Vascular Mechanobiology: Homeostasis, Adaptation, and Disease. Annu. Rev. Biomed. Eng..

[28] Jorba I., Mostert D., Hermans L.H., van der Pol A., Kurniawan N.A., Bouten C.V. (2021). In Vitro Methods to Model Cardiac Mechanobiology in Health and Disease. Tissue Eng. Part C Methods.

[29] Yadav S., Ta H.T., Nguyen N. (2021). Mechanobiology in cardiology: Micro- and nanotechnologies to probe mechanosignaling. VIEW.

[30] Garoffolo G., Pesce M. (2019). Mechanotransduction in the Cardiovascular System: From Developmental Origins to Homeostasis and Pathology. Cells.

[31] Jabre S., Hleihel W., Coirault C. (2021). Nuclear Mechanotransduction in Skeletal Muscle. Cells.

[32] Guo X.E., Hung C.T., Sandell L.J., Silva M.J. (2018). Musculoskeletal mechanobiology: A new era for MechanoMedicine. J. Orthop. Res..

[33] van Ingen M.J.A., Kirby T.J. (2021). LINCing Nuclear Mechanobiology With Skeletal Muscle Mass and Function. Front. Cell Dev. Biol..

[34] Khuu S., Fernandez J.W., Handsfield G.G. (2021). A Coupled Mechanobiological Model of Muscle Regeneration In Cerebral Palsy. Front. Bioeng. Biotechnol..

[35] Clippinger S.R., Cloonan P.E., Greenberg L., Ernst M., Stump W.T., Greenberg M.J. (2019). Disrupted mechanobiology links the molecular and cellular phenotypes in familial dilated cardiomyopathy. Proc. Natl. Acad. Sci. USA.

[36] Barnes J.M., Przybyla L., Weaver V.M. (2017). Tissue mechanics regulate brain development, homeostasis and disease. J. Cell Sci..

[37] Marinval N., Chew S.Y. (2021). Mechanotransduction assays for neural regeneration strategies: A focus on glial cells. APL Bioeng..

[38] Procès A., Luciano M., Kalukula Y., Ris L., Gabriele S. (2022). Multiscale Mechanobiology in Brain Physiology and Diseases. Front. Cell Dev. Biol..

[39] Argentati C., Morena F., Tortorella I., Bazzucchi M., Porcellati S., Emiliani C., Martino S. (2019). Insight into Mechanobiology: How stem cells feel mechanical forces and orchestrate biobical functions. Int. J. Mol. Sci..

[40] Maurer M., Lammerding J. (2019). The Driving Force: Nuclear Mechanotransduction in Cellular Function, Fate, and Disease. Annu. Rev. Biomed. Eng..

[41] Ingber D.E. (1997). Tensegrity: The Architectural Basis of Cellular. Annu. Rev. Physiol..

[42] Ingber D.E., Wang N., Stamenovic D. (2014). Tensegrity, cellular biophysics, and the mechanics of living systems. Rep. Prog. Phys..

[43] Ingber D.E. (2018). From mechanobiology to developmentally inspired engineering. Philos. Trans. R. Soc. B Biol. Sci..

[44] Mathieu S., Manneville J.-B. (2019). Intracellular mechanics: Connecting rheology and mechanotransduction. Curr. Opin. Cell Biol..

[45] Mierke C.T. (2022). Viscoelasticity, Like Forces, Plays a Role in Mechanotransduction. Front. Cell Dev. Biol..

[46] Corominas-Murtra B., Petridou N.I. (2021). Viscoelastic Networks: Forming Cells and Tissues. Front. Phys..

[47] Uray I.P., Uray K. (2021). Mechanotransduction at the Plasma Membrane-Cytoskeleton Interface. Int. J. Mol. Sci..

[48] Lavrenyuk K., Conway D., Dahl K.N. (2021). Imaging methods in mechanosensing: A historical perspective and visions for the future. Mol. Biol. Cell.

[49] Chugh M., Munjal A., Megason S.G. (2022). Hydrostatic pressure as a driver of cell and tissue morphogenesis. Semin. Cell Dev. Biol..

[50] Jin P., Jan L.Y., Jan Y.-N. (2020). Mechanosensitive Ion Channels: Structural Features Relevant to Mechanotransduction Mechanisms. Annu. Rev. Neurosci..

[51] Swaminathan V., Gloerich M. (2021). Decoding mechanical cues by molecular mechanotransduction. Curr. Opin. Cell Biol..

[52] Qin L., He T., Chen S., Yang D., Yi W., Cao H., Xiao G. (2021). Roles of mechanosensitive channel Piezo1/2 proteins in skeleton and other tissues. Bone Res..

[53] Pathak M.M., Nourse J.L., Tran T., Hwe J., Arulmoli J., Dai Trang T.L., Bernardis E., Flanagan L.A., Tombola F. (2014). Stretch-activated ion channel Piezo1 directs lineage choice in human neural stem cells. Proc. Natl. Acad. Sci. USA.

[54] Chi S., Cui Y., Wang H., Jiang J., Zhang T., Sun S., Zhou Z., Zhong Y., Xiao B. (2022). Astrocytic Piezo1-mediated mechanotransduction determines adult neurogenesis and cognitive functions. Neuron.

[55] Alper S. (2017). Genetic Diseases of PIEZO1 and PIEZO2 Dysfunction. Curr. Top. Membr..

[56] Li X., Hu J., Zhao X., Li J., Chen Y. (2022). Piezo channels in the urinary system. Exp. Mol. Med..

[57] Velasco-Estevez M., Gadalla K., Liñan-Barba N., Cobb S., Dev K.K., Sheridan G.K. (2020). Inhibition of Piezo1 attenuates demyelination in the central nervous system. Glia.

[58] Lai A., Cox C.D., Sekar N.C., Thurgood P., Jaworowski A., Peter K., Baratchi S. (2022). Mechanosensing by Piezo1 and its implications for physiology and various pathologies. Biol. Rev..

[59] Yang K., He X., Wu Z., Yin Y., Pan H., Zhao X., Sun T. (2022). The emerging roles of piezo1 channels in animal models of multiple sclerosis. Front. Immunol..

[60] Zhu W., Guo S., Homilius M., Nsubuga C., Wright S.H., Quan D., Kc A., Eddy S.S., Victorio R.A., Beerens M. (2022). PIEZO1 mediates a mechanothrombotic pathway in diabetes. Sci. Transl. Med..

[61] Sforna L., Michelucci A., Morena F., Argentati C., Franciolini F., Vassalli M., Martino S., Catacuzzeno L. (2022). Piezo1 controls cell volume and migration by modulating swelling-activated chloride current through Ca^2+^ influx. J. Cell. Physiol..

[62] Gaub B.M., Müller D.J. (2017). Mechanical Stimulation of Piezo1 Receptors Depends on Extracellular Matrix Proteins and Directionality of Force. Nano Lett..

[63] Retailleau K., Duprat F., Arhatte M., Ranade S.S., Peyronnet R., Martins J.R., Jodar M., Moro C., Offermanns S., Feng Y. (2015). Piezo1 in Smooth Muscle Cells Is Involved in Hypertension-Dependent Arterial Remodeling. Cell Rep..

[64] Ridone P., Vassalli M., Martinac B. (2019). Piezo1 mechanosensitive channels: What are they and why are they important. Biophys. Rev..

[65] Chuang Y.-C., Chen C.-C. (2022). Force From Filaments: The Role of the Cytoskeleton and Extracellular Matrix in the Gating of Mechanosensitive Channels. Front. Cell Dev. Biol..

[66] Wang J., Jiang J., Yang X., Zhou G., Wang L., Xiao B. (2022). Tethering Piezo channels to the actin cytoskeleton for mechanogating via the cadherin-β-catenin mechanotransduction complex. Cell Rep..

[67] Wiedmann F., Rinné S., Donner B., Decher N., Katus H.A., Schmidt C. (2020). Mechanosensitive TREK-1 two-pore-domain potassium (K2P) channels in the cardiovascular system. Prog. Biophys. Mol. Biol..

[68] Yap A.S., Duszyc K., Viasnoff V. (2017). Mechanosensing and Mechanotransduction at Cell–Cell Junctions. Cold Spring Harb. Perspect. Biol..

[69] Cai X., Wang K.-C., Meng Z. (2021). Mechanoregulation of YAP and TAZ in Cellular Homeostasis and Disease Progression. Front. Cell Dev. Biol..

[70] Martino F., Perestrelo A.R., Vinarský V., Pagliari S., Forte G. (2018). Cellular mechanotransduction: From tension to function. Front. Physiol..

[71] Astudillo P. (2020). Extracellular matrix stiffness and Wnt/β-catenin signaling in physiology and disease. Biochem. Soc. Trans..

[72] Amit C., Padmanabhan P., Narayanan J. (2020). Deciphering the mechanoresponsive role of β-catenin in keratoconus epithelium. Sci. Rep..

[73] Yu F., Yu C., Li F., Zuo Y., Wang Y., Yao L., Wu C., Wang C., Ye L. (2021). Wnt/β-catenin signaling in cancers and targeted therapies. Signal Transduct. Target. Ther..

[74] del Río Hernández A., Warboys C.M. (2018). Mechanoactivation of Wnt/β-catenin pathways in health and disease. Emerg. Top. Life Sci..

[75] Ma X., Hammes S.R. (2018). Paxillin actions in the nucleus. Steroids.

[76] Noh K., Bach D.-H., Choi H.-J., Kim M.S., Wu S.Y., Pradeep S., Ivan C., Cho M.-S., Bayraktar E., Rodriguez-Aguayo C. (2020). The hidden role of paxillin: Localization to nucleus promotes tumor angiogenesis. Oncogene.

[77] Silva A.J.D., Hästbacka H.S.E., Puustinen M.C., Pessa J.C., Goult B.T., Jacquemet G., Henriksson E., Sistonen L. (2022). A subpopulation of Talin 1 resides in the nucleus and regulates gene expression. bioRxiv.

[78] LeBlanc L., Ramirez N., Kim J. (2021). Context-dependent roles of YAP/TAZ in stem cell fates and cancer. Cell. Mol. Life Sci..

[79] He L., Pratt H., Gao M., Wei F., Weng Z., Struhl K. (2021). YAP and TAZ are transcriptional co-activators of AP-1 proteins and STAT3 during breast cellular transformation. eLife.

[80] Dupont S. (2018). Regulation of YAP/TAZ Activity by Mechanical Cues: An Experimental Overview. Methods Mol. Biol..

[81] Burridge K., Monaghan-Benson E., Graham D.M. (2019). Mechanotransduction: From the cell surface to the nucleus via RhoA. Philos. Trans. R. Soc. B Biol. Sci..

[82] Crosas-Molist E., Samain R., Kohlhammer L., Orgaz J.L., George S.L., Maiques O., Barcelo J., Sanz-Moreno V. (2022). Rho GTPase signaling in cancer progression and dissemination. Physiol. Rev..

[83] Boyle S.T., Kular J., Nobis M., Ruszkiewicz A., Timpson P., Samuel M.S. (2020). Acute compressive stress activates RHO/ROCK-mediated cellular processes. Small GTPases.

[84] Totaro A., Zhuang Q., Panciera T., Battilana G., Azzolin L., Brumana G., Gandin A., Brusatin G., Cordenonsi M., Piccolo S. (2019). Cell phenotypic plasticity requires autophagic flux driven by YAP/TAZ mechanotransduction. Proc. Natl. Acad. Sci. USA.

[85] Boyko S., Surewicz W.K. (2022). Tau liquid–liquid phase separation in neurodegenerative diseases. Trends Cell Biol..

[86] Wiersma V., Rigort R., Polymenidou M. (2022). Tau: A phase in the crowd. EMBO J..

[87] Kanekura K., Kuroda M. (2022). How can we interpret the relationship between liquid-liquid phase separation and amyotrophic lateral sclerosis?. Lab. Investig..

[88] Carey J.L., Guo L. (2022). Liquid-Liquid Phase Separation of TDP-43 and FUS in Physiology and Pathology of Neurodegenerative Diseases. Front. Mol. Biosci..

[89] Zbinden A., Pérez-Berlanga M., De Rossi P., Polymenidou M. (2020). Phase Separation and Neurodegenerative Diseases: A Disturbance in the Force. Dev. Cell.

[90] Morrow C.S., Porter T.J., Xu N., Arndt Z.P., Ako-Asare K., Heo H., Thompson E.A., Moore D.L. (2020). Vimentin Coordinates Protein Turnover at the Aggresome during Neural Stem Cell Quiescence Exit. Cell Stem Cell.

[91] Feng Q., Kornmann B. (2018). Mechanical forces on cellular organelles. J. Cell Sci..

[92] Phuyal S., Baschieri F. (2020). Endomembranes: Unsung Heroes of Mechanobiology?. Front. Bioeng. Biotechnol..

[93] Zhang S., Zhao J., Quan Z., Li H., Qing H. (2022). Mitochondria and Other Organelles in Neural Development and Their Potential as Therapeutic Targets in Neurodegenerative Diseases. Front. Neurosci..

[94] Lu M., Ward E., van Tartwijk F.W., Kaminski C.F. (2021). Advances in the study of organelle interactions and their role in neurodegenerative diseases enabled by super-resolution microscopy. Neurobiol. Dis..

[95] Lee N.S., Yoon C.W., Wang Q., Moon S., Koo K.M., Jung H., Chen R., Jiang L., Lu G., Fernandez A. (2020). Focused Ultrasound Stimulates ER Localized Mechanosensitive PANNEXIN-1 to Mediate Intracellular Calcium Release in Invasive Cancer Cells. Front. Cell Dev. Biol..

[96] Nava M., Miroshnikova Y.A., Biggs L., Whitefield D.B., Metge F., Boucas J., Vihinen H., Jokitalo E., Li X., Arcos J.M.G. (2020). Heterochromatin-Driven Nuclear Softening Protects the Genome against Mechanical Stress-Induced Damage. Cell.

[97] Singh V., Erady C., Balasubramanian N. (2018). Cell-matrix adhesion controls Golgi organization and function through Arf1 activation in anchorage-dependent cells. J. Cell Sci..

[98] Rafiq N.B.M., Nishimura Y., Plotnikov S.V., Thiagarajan V., Zhang Z., Shi S., Natarajan M., Viasnoff V., Kanchanawong P., Jones G.E. (2019). A mechano-signalling network linking microtubules, myosin IIA filaments and integrin-based adhesions. Nat. Mater..

[99] Romani P., Brian I., Santinon G., Pocaterra A., Audano M., Pedretti S., Mathieu S., Forcato M., Bicciato S., Manneville J.-B. (2019). Extracellular matrix mechanical cues regulate lipid metabolism through Lipin-1 and SREBP. Nat. Cell Biol..

[100] Romani P., Valcarcel-Jimenez L., Frezza C., Dupont S. (2020). Crosstalk between mechanotransduction and metabolism. Nat. Rev. Mol. Cell Biol..

[101] Elia I., Broekaert D., Christen S., Boon R., Radaelli E., Orth M., Verfaillie C., Grünewald T.G.P., Fendt S.-M. (2017). Proline metabolism supports metastasis formation and could be inhibited to selectively target metastasizing cancer cells. Nat. Commun..

[102] Hawk M.A., Gorsuch C.L., Fagan P., Lee C., Kim S.E., Hamann J.C., Mason J.A., Weigel K.J., Tsegaye M.A., Shen L. (2018). RIPK1-mediated induction of mitophagy compromises the viability of extracellular-matrix-detached cells. Nat. Cell Biol..

[103] Helle S.C.J., Feng Q., Aebersold M.J., Hirt L., Grüter R.R., Vahid A., Sirianni A., Mostowy S., Snedeker J.G., Šarić A. (2017). Mechanical force induces mitochondrial fission. eLife.

[104] Mahecic D., Carlini L., Kleele T., Colom A., Goujon A., Matile S., Roux A., Manley S. (2021). Mitochondrial membrane tension governs fission. Cell Rep..

[105] Shi X., Qiu H. (2022). New Insights Into Energy Substrate Utilization and Metabolic Remodeling in Cardiac Physiological Adaption. Front. Physiol..

[106] Lopaschuk G.D., Karwi Q.G., Tian R., Wende A.R., Abel E.D. (2021). Cardiac Energy Metabolism in Heart Failure. Circ. Res..

[107] Tharp K.M., Higuchi-Sanabria R., Timblin G.A., Ford B., Garzon-Coral C., Schneider C., Muncie J.M., Stashko C., Daniele J.R., Moore A.S. (2021). Adhesion-mediated mechanosignaling forces mitohormesis. Cell Metab..

[108] Bevere M., Morabito C., Mariggiò M.A., Guarnieri S. (2022). The Oxidative Balance Orchestrates the Main Keystones of the Functional Activity of Cardiomyocytes. Oxid. Med. Cell. Longev..

[109] Liao H., Qi Y., Ye Y., Yue P., Zhang D., Li Y. (2021). Mechanotranduction Pathways in the Regulation of Mitochondrial Homeostasis in Cardiomyocytes. Front. Cell Dev. Biol..

[110] Pavel M., Renna M., Park S.J., Menzies F.M., Ricketts T., Füllgrabe J., Ashkenazi A., Frake R.A., Lombarte A.C., Bento C.F. (2018). Contact inhibition controls cell survival and proliferation via YAP/TAZ-autophagy axis. Nat. Commun..

[111] Stürner E., Behl C. (2017). The Role of the Multifunctional BAG3 Protein in Cellular Protein Quality Control and in Disease. Front. Mol. Neurosci..

[112] Kirk J.A., Cheung J.Y., Feldman A.M. (2021). Therapeutic targeting of BAG3: Considering its complexity in cancer and heart disease. J. Clin. Investig..

[113] Lin H., Deaton C.A., Johnson G.V. (2022). Commentary: BAG3 as a Mediator of Endosome Function and Tau Clearance. Neuroscience.

[114] Basu S., Singh M., Verma M., Rachana R., Uddin M., Ashraf G. (2020). Multifarious Role of BAG3 in Neurodegenerative Disorders. Quality Control of Cellular Protein in Neurodegenerative Disorders.

[115] Gamerdinger M., Kaya A.M., Wolfrum U., Clement A.M., Behl C. (2011). BAG3 mediates chaperone-based aggresome-targeting and selective autophagy of misfolded proteins. EMBO Rep..

[116] Agarwal R., Paulo J.A., Toepfer C.N., Ewoldt J.K., Sundaram S., Chopra A., Zhang Q., Gorham J., DePalma S.R., Chen C.S. (2021). Filamin C Cardiomyopathy Variants Cause Protein and Lysosome Accumulation. Circ. Res..

[117] Thottacherry J.J., Kosmalska A.J., Kumar A., Vishen A.S., Elosegui-Artola A., Pradhan S., Sharma S., Singh P.P., Guadamillas M.C., Chaudhary N. (2018). Mechanochemical feedback control of dynamin independent endocytosis modulates membrane tension in adherent cells. Nat. Commun..

[118] Molnár M., Sőth Á., Simon-Vecsei Z. (2022). Pathways of integrins in the endo-lysosomal system. Biol. Futur..

[119] Shang X., Böker K.O., Taheri S., Lehmann W., Schilling A.F. (2021). Extracellular Vesicles Allow Epigenetic Mechanotransduction between Chondrocytes and Osteoblasts. Int. J. Mol. Sci..

[120] Scerra G., De Pasquale V., Pavone L.M., Caporaso M.G., Mayer A., Renna M., D’Agostino M. (2021). Early onset effects of single substrate accumulation recapitulate major features of LSD in patient-derived lysosomes. iScience.

[121] Wang G., Nola S., Bovio S., Bun P., Coppey-Moisan M., Lafont F., Galli T. (2018). Biomechanical Control of Lysosomal Secretion Via the VAMP7 Hub: A Tug-of-War between VARP and LRRK1. iScience.

[122] Herrmann M., Engelke K., Ebert R., Müller-Deubert S., Rudert M., Ziouti F., Jundt F., Felsenberg D., Jakob F. (2020). Interactions between Muscle and Bone—Where Physics Meets Biology. Biomolecules.

[123] Kamienieva I., Duszyński J., Szczepanowska J. (2021). Multitasking guardian of mitochondrial quality: Parkin function and Parkinson’s disease. Transl. Neurodegener..

[124] Bartolák-Suki E., Imsirovic J., Nishibori Y., Krishnan R., Suki B. (2017). Regulation of Mitochondrial Structure and Dynamics by the Cytoskeleton and Mechanical Factors. Int. J. Mol. Sci..

[125] Greenwell A.A., Gopal K., Ussher J.R. (2020). Myocardial Energy Metabolism in Non-ischemic Cardiomyopathy. Front. Physiol..

[126] Kashihara T., Sadoshima J. (2019). Role of YAP/TAZ in Energy Metabolism in the Heart. J. Cardiovasc. Pharmacol..

[127] Malvezzi C.C., Cabassi A., Miragoli M. (2020). Mitochondrial mechanosensor in cardiovascular diseases. Vasc. Biol..

[128] Saftig P., Puertollano R. (2021). How Lysosomes Sense, Integrate, and Cope with Stress. Trends Biochem. Sci..

[129] Zatyka M., Sarkar S., Barrett T. (2020). Autophagy in Rare (NonLysosomal) Neurodegenerative Diseases. J. Mol. Biol..

[130] Klionsky D.J., Petroni G., Amaravadi R.K., Baehrecke E.H., Ballabio A., Boya P., Pedro J.M.B., Cadwell K., Cecconi F., Choi A.M.K. (2021). Autophagy in major human diseases. EMBO J..

[131] Bonam S.R., Wang F., Muller S. (2019). Lysosomes as a therapeutic target. Nat. Rev. Drug Discov..

[132] Wallings R.L., Humble S.W., Ward M.E., Wade-Martins R. (2019). Lysosomal Dysfunction at the Centre of Parkinson’s Disease and Frontotemporal Dementia/Amyotrophic Lateral Sclerosis. Trends Neurosci..

[133] Root J., Merino P., Nuckols A., Johnson M., Kukar T. (2021). Lysosome dysfunction as a cause of neurodegenerative diseases: Lessons from frontotemporal dementia and amyotrophic lateral sclerosis. Neurobiol. Dis..

[134] Zhang W., Xu C., Sun J., Shen H.-M., Wang J., Yang C. (2022). Impairment of the autophagy–lysosomal pathway in Alzheimer’s diseases: Pathogenic mechanisms and therapeutic potential. Acta Pharm. Sin. B.

[135] Darios F., Stevanin G. (2020). Impairment of Lysosome Function and Autophagy in Rare Neurodegenerative Diseases. J. Mol. Biol..

[136] Tiribuzi R., Orlacchio A., Crispoltoni L., Maiotti M., Zampolini M., De Angeliz M., Mecocci P., Cecchetti R., Bernardi G., Datti A. (2011). Lysosomal β-Galactosidase and β-Hexosaminidase Activities Correlate with Clinical Stages of Dementia Associated with Alzheimer’s Disease and Type 2 Diabetes Mellitus. J. Alzheimer Dis..

[137] Martino S., Emiliani C., Tancini B., Severini G.M., Chigorno V., Bordignon C., Sonnino S., Orlacchio A. (2002). Absence of Metabolic Cross-correction in Tay-Sachs Cells: Implications for gene therapy. J. Biol. Chem..

[138] Santambrogio S., Ricca A., Maderna C., Ieraci A., Aureli M., Sonnino S., Kulik W., Aimar P., Bonfanti L., Martino S. (2012). The galactocerebrosidase enzyme contributes to maintain a functional neurogenic niche during early post-natal CNS development. Hum. Mol. Genet..

[139] Frati G., Luciani M., Meneghini V., De Cicco S., Ståhlman M., Blomqvist M., Grossi S., Filocamo M., Morena F., Menegon A. (2018). Human iPSC-based models highlight defective glial and neuronal differentiation from neural progenitor cells in metachromatic leukodystrophy. Cell Death Dis..

[140] Meneghini V., Lattanzi A., Tiradani L., Bravo G., Morena F., Sanvito F., Calabria A., Bringas J., Fisher-Perkins J.M., Dufour J.P. (2016). Pervasive supply of therapeutic lysosomal enzymes in the CNS of normal and Krabbe-affected non-human primates by intracerebral lentiviral gene therapy. EMBO Mol. Med..

[141] Martino S., di Girolamo I., Cavazzin C., Tiribuzi R., Galli R., Rivaroli A., Valsecchi M., Sandhoff K., Sonnino S., Vescovi A. (2009). Neural precursor cell cultures from GM2 gangliosidosis animal models recapitulate the biochemical and molecular hallmarks of the brain pathology. J. Neurochem..

[142] Fumagalli F., Calbi V., Sora M.G.N., Sessa M., Baldoli C., Rancoita P.M.V., Ciotti F., Sarzana M., Fraschini M., Zambon A.A. (2022). Lentiviral haematopoietic stem-cell gene therapy for early-onset metachromatic leukodystrophy: Long-term results from a non-randomised, open-label, phase 1/2 trial and expanded access. Lancet.

[143] Parenti G., Medina D.L., Ballabio A. (2021). The rapidly evolving view of lysosomal storage diseases. EMBO Mol. Med..

[144] Lakpa K.L., Khan N., Afghah Z., Chen X., Geiger J.D. (2021). Lysosomal Stress Response (LSR): Physiological Importance and Pathological Relevance. J. Neuroimmune Pharmacol..

[145] Malik B.R., Maddison D.C., Smith G.A., Peters O.M. (2019). Autophagic and endo-lysosomal dysfunction in neurodegenerative disease. Mol. Brain.

[146] Joseph J.G., Liu A.P. (2020). Mechanical Regulation of Endocytosis: New Insights and Recent Advances. Adv. Biosyst..

[147] Franze K., Janmey P.A., Guck J. (2013). Mechanics in Neuronal Development and Repair. Annu. Rev. Biomed. Eng..

[148] Oliveri H., Goriely A. (2022). Mathematical models of neuronal growth. Biomech. Model. Mechanobiol..

[149] Franze K. (2020). Integrating Chemistry and Mechanics: The Forces Driving Axon Growth. Annu. Rev. Cell Dev. Biol..

[150] Zhang C., Liu C., Zhao H. (2021). Mechanical properties of brain tissue based on microstructure. J. Mech. Behav. Biomed. Mater..

[151] Chighizola M., Dini T., Lenardi C., Milani P., Podestà A., Schulte C. (2019). Mechanotransduction in neuronal cell development and functioning. Biophys. Rev..

[152] Long K.R., Huttner W.B. (2019). How the extracellular matrix shapes neural development. Open Biol..

[153] Segel M., Neumann B., Hill M.F.E., Weber I.P., Viscomi C., Zhao C., Young A., Agley C.C., Thompson A.J., Gonzalez G.A. (2019). Niche stiffness underlies the ageing of central nervous system progenitor cells. Nature.

[154] Babu P.K.V., Radmacher M. (2019). Mechanics of Brain Tissues Studied by Atomic Force Microscopy: A Perspective. Front. Neurosci..

[155] Roth J.G., Huang M.S., Li T.L., Feig V.R., Jiang Y., Cui B., Greely H.T., Bao Z., Paşca S.P., Heilshorn S.C. (2021). Advancing models of neural development with biomaterials. Nat. Rev. Neurosci..

[156] Ławkowska K., Pokrywczyńska M., Koper K., Kluth L.A., Drewa T., Adamowicz J. (2021). Application of Graphene in Tissue Engineering of the Nervous System. Int. J. Mol. Sci..

[157] Kothapalli C., Mahajan G., Farrell K. (2020). Substrate stiffness induced mechanotransduction regulates temporal evolution of human fetal neural progenitor cell phenotype, differentiation, and biomechanics. Biomater. Sci..

[158] Shafiee A., Ahmadi H., Taheri B., Hosseinzadeh S., Fatahi Y., Soleimani M., Atyabi F., Dinarvand R. (2020). Appropriate Scaffold Selection for CNS Tissue Engineering. Avicenna J. Med. Biotechnol..

[159] Linka K., Reiter N., Würges J., Schicht M., Bräuer L., Cyron C.J., Paulsen F., Budday S. (2021). Unraveling the Local Relation Between Tissue Composition and Human Brain Mechanics Through Machine Learning. Front. Bioeng. Biotechnol..

[160] Menichetti A., Bartsoen L., Depreitere B., Sloten J.V., Famaey N. (2021). A Machine Learning Approach to Investigate the Uncertainty of Tissue-Level Injury Metrics for Cerebral Contusion. Front. Bioeng. Biotechnol..

[161] Schroder A., Lawrence T., Voets N., Garcia-Gonzalez D., Jones M., Peña J.-M., Jerusalem A. (2021). A Machine Learning Enhanced Mechanistic Simulation Framework for Functional Deficit Prediction in TBI. Front. Bioeng. Biotechnol..

[162] Sack I., Beierbach B., Wuerfel J., Klatt D., Hamhaber U., Papazoglou S., Martus P., Braun J. (2009). The impact of aging and gender on brain viscoelasticity. NeuroImage.

[163] Arani A., Murphy M.C., Glaser K.J., Manduca A., Lake D.S., Kruse S.A., Jack C.R., Ehman R.L., Huston J. (2015). Measuring the effects of aging and sex on regional brain stiffness with MR elastography in healthy older adults. NeuroImage.

[164] Takamura T., Motosugi U., Sasaki Y., Rt T.K., Sato K., Glaser K.J., Ehman R.L., Onishi H. (2019). Influence of Age on Global and Regional Brain Stiffness in Young and Middle-Aged Adults. J. Magn. Reson. Imaging.

[165] Yin Z., Romano A.J., Manduca A., Ehman R.L., Huston J. (2018). Stiffness and Beyond: What MR Elastography Can Tell Us About Brain Structure and Function Under Physiologic and Pathologic Conditions. Top. Magn. Reson. Imaging.

[166] Hiscox L.V., Schwarb H., McGarry M.D., Johnson C.L. (2021). Aging brain mechanics: Progress and promise of magnetic resonance elastography. NeuroImage.

[167] Delgorio P.L., Hiscox L.V., Daugherty A.M., Sanjana F., Pohlig R.T., Ellison J.M., Martens C.R., Schwarb H., McGarry M.D.J., Johnson C.L. (2021). Effect of Aging on the Viscoelastic Properties of Hippocampal Subfields Assessed with High-Resolution MR Elastography. Cereb. Cortex.

[168] Ngo M.T., Harley B.A.C. (2021). Progress in mimicking brain microenvironments to understand and treat neurological disorders. APL Bioeng..

[169] Chatterjee K., Carman-Esparza C.M., Munson J.M. (2019). Methods to measure, model and manipulate fluid flow in brain. J. Neurosci. Methods.

[170] Cobbaut M., Karagil S., Bruno L., Loza M.D.C.D.D.L., Mackenzie F., Stolinski M., Elbediwy A. (2020). Dysfunctional Mechanotransduction through the YAP/TAZ/Hippo Pathway as a Feature of Chronic Disease. Cells.

[171] Muñoz-Lasso D.C., Romá-Mateo C., Pallardó F.V., Gonzalez-Cabo P. (2020). Much More Than a Scaffold: Cytoskeletal Proteins in Neurological Disorders. Cells.

[172] Gharaba S., Paz O., Feld L., Abashidze A., Weinrab M., Wolfenson H., Weil M. (2022). Perturbed actin cap and nuclear morphology in primary fibroblasts of Huntington’s disease patients as a new phenotypic marker for personalized drug evaluation. bioRxiv.

[173] Castellanos-Montiel M.J., Chaineau M., Durcan T.M. (2020). The Neglected Genes of ALS: Cytoskeletal Dynamics Impact Synaptic Degeneration in ALS. Front. Cell. Neurosci..

[174] Rush T., Martinez-Hernandez J., Dollmeyer M., Frandemiche M.L., Borel E., Boisseau S., Jacquier-Sarlin M., Buisson A. (2018). Synaptotoxicity in Alzheimer’s Disease Involved a Dysregulation of Actin Cytoskeleton Dynamics through Cofilin 1 Phosphorylation. J. Neurosci..

[175] Wang Q., Yuan W., Yang X., Wang Y., Li Y., Qiao H. (2020). Role of Cofilin in Alzheimer’s Disease. Front. Cell Dev. Biol..

[176] Kang D.E., Woo J.A. (2019). Cofilin, a Master Node Regulating Cytoskeletal Pathogenesis in Alzheimer’s Disease. J. Alzheimers. Dis..

[177] Lapeña-Luzón T., Rodríguez L.R., Beltran-Beltran V., Benetó N., Pallardó F.V., Gonzalez-Cabo P. (2021). Cofilin and Neurodegeneration: New Functions for an Old but Gold Protein. Brain Sci..

[178] Schmidt S.I., Blaabjerg M., Freude K., Meyer M. (2022). RhoA Signaling in Neurodegenerative Diseases. Cells.

[179] Oliveira da Silva M.I., Liz M.A. (2020). Linking Alpha-Synuclein to the Actin Cytoskeleton: Consequences to Neuronal Function. Front. Cell Dev. Biol..

[180] Richardson D., McEntagart M.M., Isaacs J.D. (2020). DCTN1-related Parkinson-plus disorder (Perry syndrome). Pract. Neurol..

[181] Burns L.H., Wang H.-Y. (2017). Altered filamin A enables amyloid beta-induced tau hyperphosphorylation and neuroinflammation in Alzheimer’s disease. Neuroimmunol. Neuroinflamm..

[182] Seervai R.N.H., Jangid R.K., Karki M., Tripathi D.N., Jung S.Y., Kearns S.E., Verhey K.J., Cianfrocco M.A., Millis B.A., Tyska M.J. (2020). The Huntingtin-interacting protein SETD2/HYPB is an actin lysine methyltransferase. Sci. Adv..

[183] Dourlen P., Kilinc D., Malmanche N., Chapuis J., Lambert J.C. (2019). The new genetic landscape of Alzheimer’s disease: From amyloid cascade to genetically driven synaptic failure hypothesis?. Acta Neuropathol..

[184] Eysert F., Coulon A., Boscher E., Vreulx A.-C., Flaig A., Mendes T., Hughes S., Grenier-Boley B., Hanoulle X., Demiautte F. (2020). Alzheimer’s genetic risk factor FERMT2 (Kindlin-2) controls axonal growth and synaptic plasticity in an APP-dependent manner. Mol. Psychiatry.

[185] Nicolas A., Kenna K.P., Renton A.E., Ticozzi N., Faghri F., Chia R., Dominov J.A., Kenna B.J., Nalls M.A., Keagle P. (2018). Genome-wide analyses identify KIF5A as a novel ALS gene. Neuron.

[186] Zhang S., Cooper-Knock J., Weimer A.K., Shi M., Moll T., Marshall J.N., Harvey C., Nezhad H.G., Franklin J., Souza C.D.S. (2022). Genome-wide identification of the genetic basis of amyotrophic lateral sclerosis. Neuron.

[187] Gil L., Niño S.A., Capdeville G., Jiménez-Capdeville M.E. (2021). Aging and Alzheimer’s disease connection: Nuclear Tau and lamin A. Neurosci. Lett..

[188] Alcalá-Vida R., Garcia-Forn M., Castany-Pladevall C., Creus-Muncunill J., Ito Y., Blanco E., Golbano A., Crespí-Vázquez K., Parry A., Slater G. (2020). Neuron type-specific increase in lamin B1 contributes to nuclear dysfunction in Huntington’s disease. EMBO Mol. Med..

[189] Matias I., Diniz L.P., Damico I.V., Araujo A.P.B., Neves L.D.S., Vargas G., Leite R.E.P., Suemoto C.K., Nitrini R., Jacob-Filho W. (2021). Loss of lamin-B1 and defective nuclear morphology are hallmarks of astrocyte senescence in vitro and in the aging human hippocampus. Aging Cell.

[190] Gutiérrez-Vargas J., Castro-Álvarez J., Zapata-Berruecos J., Abdul-Rahim K., Arteaga-Noriega A. (2022). Neurodegeneration and convergent factors contributing to the deterioration of the cytoskeleton in Alzheimer’s disease, cerebral ischemia and multiple sclerosis (Review). Biomed. Rep..

[191] DeGiosio R.A., Grubisha M.J., MacDonald M.L., McKinney B.C., Camacho C.J., Sweet R.A. (2022). More than a marker: Potential pathogenic functions of MAP2. Front. Mol. Neurosci..

[192] Cabrera J.R., Lucas J.J. (2016). MAP2 Splicing is Altered in Huntington’s Disease. Brain Pathol..

[193] Oeckl P., Weydt P., Thal D.R., Weishaupt J.H., Ludolph A.C., Otto M. (2019). Proteomics in cerebrospinal fluid and spinal cord suggests UCHL1, MAP2 and GPNMB as biomarkers and underpins importance of transcriptional pathways in amyotrophic lateral sclerosis. Acta Neuropathol..

[194] Varga B., Martin-Fernandez M., Hilaire C., Sanchez-Vicente A., Areias J., Salsac C., Cuisinier F.J.G., Raoul C., Scamps F., Gergely C. (2018). Myotube elasticity of an amyotrophic lateral sclerosis mouse model. Sci. Rep..

[195] Jun M.-H., Jang J.-W., Jeon P., Lee S.-K., Lee S.-H., Choi H.-E., Lee Y.-K., Choi H., Park S.-W., Kim J. (2020). Nonmuscle myosin IIB regulates Parkin-mediated mitophagy associated with amyotrophic lateral sclerosis-linked TDP. Cell Death Dis..

[196] Hu J., Lin S.L., Schachner M. (2022). A fragment of cell adhesion molecule L1 reduces amyloid-β plaques in a mouse model of Alzheimer’s disease. Cell Death Dis..

[197] Chou C.-C., Zhang Y., Umoh M.E., Vaughan S.W., Lorenzini I., Liu F., Sayegh M., Donlin-Asp P., Chen Y.H., Duong D. (2018). TDP-43 pathology disrupts nuclear pore complexes and nucleocytoplasmic transport in ALS/FTD. Nat. Neurosci..

[198] Fallini C., Khalil B., Smith C.L., Rossoll W. (2020). Traffic jam at the nuclear pore: All roads lead to nucleocytoplasmic transport defects in ALS/FTD. Neurobiol. Dis..

[199] Velasco M., Mampay M., Boutin H., Chaney A., Warn P., Sharp A., Burgess E., Moeendarbary E., Dev K.K., Sheridan G.K. (2018). Infection Augments Expression of Mechanosensing Piezo1 Channels in Amyloid Plaque-Reactive Astrocytes. Front. Aging Neurosci..

[200] Velasco M., Rolle S.O., Mampay M., Dev K.K., Sheridan G.K. (2019). Piezo1 regulates calcium oscillations and cytokine release from astrocytes. Glia.

[201] Ordonez D.G., Lee M.K., Feany M.B. (2017). α-synuclein Induces Mitochondrial Dysfunction through Spectrin and the Actin Cytoskeleton. Neuron.

[202] Cappelletti G., Calogero A.M., Rolando C. (2021). Microtubule acetylation: A reading key to neural physiology and degeneration. Neurosci. Lett..

[203] Billon C., Adham S., Poblete N.H., Legrand A., Frank M., Chiche L., Zuily S., Benistan K., Savale L., Zaafrane-Khachnaoui K. (2021). Cardiovascular and connective tissue disorder features in FLNA-related PVNH patients: Progress towards a refined delineation of this syndrome. Orphanet J. Rare Dis..

[204] Sasaki E., Byrne A.T., Phelan E., Cox D.W., Reardon W. (2018). A review of filamin A mutations and associated interstitial lung disease. Eur. J. Pediatr..

[205] Castaño-Jaramillo L.M., Lugo-Reyes S.O., Cruz Muñoz M.E., Scheffler-Mendoza S.C., Duran McKinster C., Yamazaki-Nakashimada M.A., Espinosa-Padilla S.E., Saez-de-Ocariz Gutierrez M.d.M. (2021). Diagnostic and therapeutic caveats in Griscelli syndrome. Scand. J. Immunol..

[206] Griscelli Syndrome Type 1 (Concept Id: C1859194)-MedGen-NCBI. https://www.ncbi.nlm.nih.gov/medgen/347092.

[207] Vontell R., Supramaniam V.G., Davidson A., Thornton C., Marnerides A., Holder-Espinasse M., Lillis S., Yau S., Jansson M., Hagberg H.E. (2019). Post-mortem Characterisation of a Case With an ACTG1 Variant, Agenesis of the Corpus Callosum and Neuronal Heterotopia. Front. Physiol..

[208] Parker F., Baboolal T.G., Peckham M. (2020). Actin Mutations and Their Role in Disease. Int. J. Mol. Sci..

[209] Argentati C., Tortorella I., Bazzucchi M., Emiliani C., Morena F., Martino S. (2020). The Other Side of Alzheimer’s Disease: Influence of Metabolic Disorder Features for Novel Diagnostic Biomarkers. J. Pers. Med..

[210] Gerischer L.M., Fehlner A., Köbe T., Prehn K., Antonenko D., Grittner U., Braun J., Sack I., Flöel A. (2017). Combining viscoelasticity, diffusivity and volume of the hippocampus for the diagnosis of Alzheimer’s disease based on magnetic resonance imaging. NeuroImage. Clin..

[211] Hiscox L.V., Johnson C.L., McGarry M.D.J., Marshall H., Ritchie C.W., Van Beek E.J.R., Roberts N., Starr J.M. (2019). Mechanical property alterations across the cerebral cortex due to Alzheimer’s disease. Brain Commun..

[212] Fawcett J.W., Oohashi T., Pizzorusso T. (2019). The roles of perineuronal nets and the perinodal extracellular matrix in neuronal function. Nat. Rev. Neurosci..

[213] Logsdon A.F., Francis K.L., Richardson N.E., Hu S.J., Faber C.L., Phan B.A., Nguyen V., Setthavongsack N., Banks W.A., Woltjer R.L. (2021). Decoding perineuronal net glycan sulfation patterns in the Alzheimer’s disease brain. Alzheimer Dement..

[214] Reichelt A.C. (2020). Is loss of perineuronal nets a critical pathological event in Alzheimer’s disease?. EBioMedicine.

[215] Lu Z., Li H., Hou C., Peng Y., Long J., Liu J. (2016). Endogenously generated amyloid-β increases stiffness in human neuroblastoma cells. Eur. Biophys. J..

[216] Bruno L., Karagil S., Mahmood A., Elbediwy A., Stolinski M., Mackenzie F.E. (2021). Mechanosensing and the Hippo Pathway in Microglia: A Potential Link to Alzheimer’s Disease Pathogenesis?. Cells.

[217] Hu J., Zhu H., Yang Q., Shen H., Chai G., Zhang B., Chen S., Chen Q., Cai Z., Li X. (2022). Microglial Piezo1 senses Aβ fibrils stiffness to restrict Alzheimer’s disease. bioRxiv.

[218] Hall C.M., Moeendarbary E., Sheridan G.K. (2020). Mechanobiology of the brain in ageing and Alzheimer’s disease. Eur. J. Neurosci..

[219] Xu J., Patassini S., Rustogi N., Riba-Garcia I., Hale B.D., Phillips A.M., Waldvogel H., Haines R., Bradbury P., Stevens A. (2019). Regional protein expression in human Alzheimer’s brain correlates with disease severity. Commun. Biol..

[220] Tanaka H., Homma H., Fujita K., Kondo K., Yamada S., Jin X., Waragai M., Ohtomo G., Iwata A., Tagawa K. (2020). YAP-dependent necrosis occurs in early stages of Alzheimer’s disease and regulates mouse model pathology. Nat. Commun..

[221] Leshchyns’Ka I., Sytnyk V. (2016). Synaptic Cell Adhesion Molecules in Alzheimer’s Disease. Neural Plast..

[222] Nielsen H., Wennström M. (2012). Cell adhesion molecules in Alzheimer’s disease. Degener. Neurol. Neuromuscul. Dis..

[223] Ilic K., Mlinac-Jerković K., Jovanov-Milosevic N., Simic G., Habek N., Bogdanovic N., Kalanj-Bognar S. (2018). Hippocampal expression of cell-adhesion glycoprotein neuroplastin is altered in Alzheimer’s disease. J. Cell. Mol. Med..

[224] Kozlova I., Sah S., Keable R., Leshchyns’Ka I., Janitz M., Sytnyk V. (2020). Cell Adhesion Molecules and Protein Synthesis Regulation in Neurons. Front. Mol. Neurosci..

[225] Simufilam (PTI-125). https://alzheimersnewstoday.com/pti-125/.

[226] Simufilam|ALZFORUM. https://www.alzforum.org/therapeutics/simufilam.

[227] Wang H.-Y., Lee K.-C., Pei Z., Khan A., Bakshi K., Burns L.H. (2017). PTI-125 binds and reverses an altered conformation of filamin A to reduce Alzheimer’s disease pathogenesis. Neurobiol. Aging.

[228] Simufilam 50 mg or 100 mg for Mild-to-Moderate Alzheimer’s Disease-Full Text View-ClinicalTrials.gov. https://clinicaltrials.gov/ct2/show/NCT05026177.

[229] Simufilam 100 mg for Mild-to-Moderate Alzheimer’s Disease-Full Text View-ClinicalTrials.gov. https://clinicaltrials.gov/ct2/show/NCT04994483?term=Simufilam&draw=2&rank=2.

[230] Bamburg J.R., Minamide L.S., Wiggan O., Tahtamouni L.H., Kuhn T.B. (2021). Cofilin and Actin Dynamics: Multiple Modes of Regulation and Their Impacts in Neuronal Development and Degeneration. Cells.

[231] Namme J.N., Bepari A.K., Takebayashi H. (2021). Cofilin Signaling in the CNS Physiology and Neurodegeneration. Int. J. Mol. Sci..

[232] Gomez-Gutierrez R., Morales R. (2020). The prion-like phenomenon in Alzheimer’s disease: Evidence of pathology transmission in humans. PLoS Pathog..

[233] Condello C., DeGrado W.F., Prusiner S.B. (2020). Prion biology: Implications for Alzheimer’s disease therapeutics. Lancet Neurol..

[234] Zhang Y., Zhao Y., Zhang L., Yu W., Wang Y., Chang W. (2019). Cellular Prion Protein as a Receptor of Toxic Amyloid-β42 Oligomers Is Important for Alzheimer’s Disease. Front. Cell. Neurosci..

[235] Shafiq M., Zafar S., Younas N., Noor A., Puig B., Altmeppen H.C., Schmitz M., Matschke J., Ferrer I., Glatzel M. (2021). Prion protein oligomers cause neuronal cytoskeletal damage in rapidly progressive Alzheimer’s disease. Mol. Neurodegener..

[236] Frost B. (2016). Alzheimer’s disease: An acquired neurodegenerative laminopathy. Nucleus.

[237] Vahabikashi A., Adam S.A., Medalia O., Goldman R.D. (2022). Nuclear lamins: Structure and function in mechanobiology. APL Bioeng..

[238] Shin Y., Chang Y.-C., Lee D.S., Berry J., Sanders D.W., Ronceray P., Wingreen N.S., Haataja M., Brangwynne C.P. (2018). Liquid Nuclear Condensates Mechanically Sense and Restructure the Genome. Cell.

[239] Iatrou A., Clark E.M., Wang Y. (2021). Nuclear dynamics and stress responses in Alzheimer’s disease. Mol. Neurodegener..

[240] Gil L., Niño S.A., Chi-Ahumada E., Rodríguez-Leyva I., Guerrero C., Rebolledo A.B., Arias J.A., Jiménez-Capdeville M.E. (2020). Perinuclear Lamin A and Nucleoplasmic Lamin B2 Characterize Two Types of Hippocampal Neurons through Alzheimer’s Disease Progression. Int. J. Mol. Sci..

[241] Lin W.-Q., Ngian Z.-K., Koh T.-W., Ong C.-T. (2022). Altered stability of nuclear lamin-B marks the onset of aging in male Drosophila. PLoS ONE.

[242] Murray H.C., Johnson K., Sedlock A., Highet B., Dieriks B.V., Anekal P.V., Faull R.L.M., Curtis M.A., Koretsky A., Maric D. (2022). Lamina-specific immunohistochemical signatures in the olfactory bulb of healthy, Alzheimer’s and Parkinson’s disease patients. Commun. Biol..

[243] Jiang L., Wolozin B. (2021). Oligomeric tau disrupts nuclear envelope via binding to lamin proteins and lamin B receptor. Alzheimer Dement..

[244] Ippati S., Deng Y., van der Hoven J., Heu C., van Hummel A., Chua S.W., Paric E., Chan G., Feiten A., Fath T. (2021). Rapid initiation of cell cycle reentry processes protects neurons from amyloid-β toxicity. Proc. Natl. Acad. Sci. USA.

[245] Nandakumar S., Rozich E., Buttitta L. (2021). Cell Cycle Re-entry in the Nervous System: From Polyploidy to Neurodegeneration. Front. Cell Dev. Biol..

[246] Calzoni E., Argentati C., Cesaretti A., Montegiove N., Tortorella I., Bazzucchi M., Morena F., Martino S., Emiliani C. (2021). RNA Modifications in Neurodegenerations. Epitranscriptomics.

[247] Komatsu H. (2021). Innovative Therapeutic Approaches for Huntington’s Disease: From Nucleic Acids to GPCR-Targeting Small Molecules. Front. Cell. Neurosci..

[248] Tabrizi S.J., Flower M., Ross C.A., Wild E.J. (2020). Huntington disease: New insights into molecular pathogenesis and therapeutic opportunities. Nat. Rev. Neurol..

[249] Taran A.S., Shuvalova L.D., Lagarkova M.A., Alieva I.B. (2020). Huntington’s Disease—An Outlook on the Interplay of the HTT Protein, Microtubules and Actin Cytoskeletal Components. Cells.

[250] Maninova M., Caslavsky J., Vomastek T. (2017). The assembly and function of perinuclear actin cap in migrating cells. Protoplasma.

[251] Hardiman O., Al-Chalabi A., Chio A., Corr E.M., Logroscino G., Robberecht W., Shaw P.J., Simmons Z., Van Den Berg L.H. (2017). Amyotrophic lateral sclerosis. Nat. Rev. Dis. Prim..

[252] Masrori P., Van Damme P. (2020). Amyotrophic lateral sclerosis: A clinical review. Eur. J. Neurol..

[253] Xu X., Shen D., Gao Y., Zhou Q., Ni Y., Meng H., Shi H., Le W., Chen S., Chen S. (2021). A perspective on therapies for amyotrophic lateral sclerosis: Can disease progression be curbed?. Transl. Neurodegener..

[254] McAlary L., Chew Y.L., Lum J.S., Geraghty N.J., Yerbury J.J., Cashman N.R. (2020). Amyotrophic Lateral Sclerosis: Proteins, Proteostasis, Prions, and Promises. Front. Cell. Neurosci..

[255] Bicchi I., Morena F., Argentati C., Nodari L.R., Emiliani C., Gelati M., Vescovi A.L., Martino S. (2021). Storage of Mutant Human SOD1 in Non-Neural Cells from the Type-1 Amyotrophic Lateral Sclerosis rat^G93A^ Model Correlated with the Lysosomes’ Dysfunction. Biomedicines.

[256] Perciballi E., Bovio F., Rosati J., Arrigoni F., D’Anzi A., Lattante S., Gelati M., De Marchi F., Lombardi I., Ruotolo G. (2022). Characterization of the p.L145F and p.S135N Mutations in SOD1: Impact on the Metabolism of Fibroblasts Derived from Amyotrophic Lateral Sclerosis Patients. Antioxidants.

[257] Chen J., Bassot A., Giuliani F., Simmen T. (2021). Amyotrophic Lateral Sclerosis (ALS): Stressed by Dysfunctional Mitochondria-Endoplasmic Reticulum Contacts (MERCs). Cells.

[258] Brasil A.D.A., de Carvalho M.D.C., Gerhardt E., Queiroz D.D., Pereira M.D., Outeiro T.F., Eleutherio E.C.A. (2019). Characterization of the activity, aggregation, and toxicity of heterodimers of WT and ALS-associated mutant Sod. Proc. Natl. Acad. Sci. USA.

[259] Huang C., Yan S., Zhang Z. (2020). Maintaining the balance of TDP-43, mitochondria, and autophagy: A promising therapeutic strategy for neurodegenerative diseases. Transl. Neurodegener..

[260] Shiota T., Nagata R., Kikuchi S., Nanaura H., Matsubayashi M., Nakanishi M., Kobashigawa S., Isozumi N., Kiriyama T., Nagayama K. (2022). C9orf72-Derived Proline:Arginine Poly-Dipeptides Modulate Cytoskeleton and Mechanical Stress Response. Front. Cell Dev. Biol..

[261] Miceli M., Exertier C., Cavaglià M., Gugole E., Boccardo M., Casaluci R.R., Ceccarelli N., De Maio A., Vallone B., Deriu M.A. (2022). ALS2-Related Motor Neuron Diseases: From Symptoms to Molecules. Biology.

[262] Bercier V., Hubbard J.M., Fidelin K., Duroure K., Auer T.O., Revenu C., Wyart C., Del Bene F. (2019). Dynactin1 depletion leads to neuromuscular synapse instability and functional abnormalities. Mol. Neurodegener..

[263] Murk K., Ornaghi M., Schiweck J. (2021). Profilin Isoforms in Health and Disease–All the Same but Different. Front. Cell Dev. Biol..

[264] Poesen K., Van Damme P. (2019). Diagnostic and Prognostic Performance of Neurofilaments in ALS. Front. Neurol..

[265] Helferich A.M., Brockmann S.J., Reinders J., Deshpande D., Holzmann K., Brenner D., Andersen P.M., Petri S., Thal D.R., Michaelis J. (2018). Dysregulation of a novel miR-1825/TBCB/TUBA4A pathway in sporadic and familial ALS. Cell. Mol. Life Sci..

[266] Nguyen D.K., Thombre R., Wang J. (2018). Autophagy as a common pathway in amyotrophic lateral sclerosis. Neurosci. Lett..

[267] Huai J., Zhang Z. (2019). Structural Properties and Interaction Partners of Familial ALS-Associated SOD1 Mutants. Front. Neurol..

[268] Giampetruzzi A., Danielson E.W., Gumina V., Jeon M., Boopathy S., Brown R.H., Ratti A., Landers J.E., Fallini C. (2019). Modulation of actin polymerization affects nucleocytoplasmic transport in multiple forms of amyotrophic lateral sclerosis. Nat. Commun..

[269] Chandra S., Lusk C.P. (2022). Emerging Connections between Nuclear Pore Complex Homeostasis and ALS. Int. J. Mol. Sci..

[270] Gasset-Rosa F., Lu S., Yu H., Chen C., Melamed Z., Guo L., Shorter J., Da Cruz S., Cleveland D.W. (2019). Cytoplasmic TDP-43 De-mixing Independent of Stress Granules Drives Inhibition of Nuclear Import, Loss of Nuclear TDP-43, and Cell Death. Neuron.

[271] Ding B., Sepehrimanesh M. (2021). Nucleocytoplasmic Transport: Regulatory Mechanisms and the Implications in Neurodegeneration. Int. J. Mol. Sci..

[272] Bloem B.R., Okun M.S., Klein C. (2021). Parkinson’s disease. Lancet.

[273] Ou Z., Pan J., Tang S., Duan D., Yu D., Nong H., Wang Z. (2021). Global Trends in the Incidence, Prevalence, and Years Lived With Disability of Parkinson’s Disease in 204 Countries/Territories From 1990 to 2019. Front. Public Health.

[274] Day J., Mullin S. (2021). The Genetics of Parkinson’s Disease and Implications for Clinical Practice. Genes.

[275] Seebauer L., Schneider Y., Drobny A., Plötz S., Koudelka T., Tholey A., Prots I., Winner B., Zunke F., Winkler J. (2022). Interaction of Alpha Synuclein and Microtubule Organization Is Linked to Impaired Neuritic Integrity in Parkinson’s Patient-Derived Neuronal Cells. Int. J. Mol. Sci..

[276] Amadeo A., Pizzi S., Comincini A., Modena D., Calogero A.M., Madaschi L., Faustini G., Rolando C., Bellucci A., Pezzoli G. (2021). The Association between α-Synuclein and α-Tubulin in Brain Synapses. Int. J. Mol. Sci..

[277] Calogero A.M., Mazzetti S., Pezzoli G., Cappelletti G. (2019). Neuronal microtubules and proteins linked to Parkinson’s disease: A relevant interaction?. Biol. Chem..

[278] Casagrande F.V., Amadeo A., Cartelli D., Calogero A.M., Modena D., Costa I., Cantele F., Onelli E., Moscatelli A., Ascagni M. (2019). The imbalance between dynamic and stable microtubules underlies neurodegeneration induced by 2,5-hexanedione. Biochim. Biophys. Acta Mol. Basis Dis..

[279] Kruppa A., Kishi-Itakura C., Masters T., Rorbach J., Grice G.L., Kendrick-Jones J., Nathan J., Minczuk M., Buss F. (2018). Myosin VI-Dependent Actin Cages Encapsulate Parkin-Positive Damaged Mitochondria. Dev. Cell.

[280] Kruppa A.J., Buss F. (2021). Motor proteins at the mitochondria–cytoskeleton interface. J. Cell Sci..

[281] Jetto C.T., Nambiar A., Manjithaya R. (2022). Mitophagy and Neurodegeneration: Between the Knowns and the Unknowns. Front. Cell Dev. Biol..

[282] Zhang Q., Xu Y., Lee J., Jarnik M., Wu X., Bonifacino J.S., Shen J., Ye Y. (2020). A myosin-7B–dependent endocytosis pathway mediates cellular entry of α-synuclein fibrils and polycation-bearing cargos. Proc. Natl. Acad. Sci. USA.

[283] Martínez-Cué C., Rueda N. (2020). Cellular Senescence in Neurodegenerative Diseases. Front. Cell. Neurosci..

[284] Paonessa F., Evans L.D., Solanki R., Larrieu D., Wray S., Hardy J., Jackson S.P., Livesey F.J. (2019). Microtubules Deform the Nuclear Membrane and Disrupt Nucleocytoplasmic Transport in Tau-Mediated Frontotemporal Dementia. Cell Rep..

[285] Chinta S.J., Woods G., Demaria M., Rane A., Zou Y., McQuade A., Rajagopalan S., Limbad C., Madden D.T., Campisi J. (2018). Cellular Senescence Is Induced by the Environmental Neurotoxin Paraquat and Contributes to Neuropathology Linked to Parkinson’s Disease. Cell Rep..

[286] Iyer M., Subramaniam M.D., Venkatesan D., Cho S.G., Ryding M., Meyer M., Vellingiri B. (2021). Role of RhoA-ROCK signaling in Parkinson’s disease. Eur. J. Pharmacol..

[287] Bogetofte H., Jensen P., Okarmus J., Schmidt S.I., Agger M., Ryding M., Nørregaard P., Fenger C., Zeng X., Graakjær J. (2019). Perturbations in RhoA signalling cause altered migration and impaired neuritogenesis in human iPSC-derived neural cells with PARK2 mutation. Neurobiol. Dis..

[288] Vaidya B., Sharma S.S. (2020). Transient Receptor Potential Channels as an Emerging Target for the Treatment of Parkinson’s Disease: An Insight Into Role of Pharmacological Interventions. Front. Cell Dev. Biol..

[289] Bella E.D., Bersano E., Antonini G., Borghero G., Capasso M., Caponnetto C., Chiò A., Corbo M., Filosto M., Giannini F. (2021). The unfolded protein response in amyotrophic later sclerosis: Results of a phase 2 trial. Brain.

[290] Malik R., Wiedau M. (2020). Therapeutic Approaches Targeting Protein Aggregation in Amyotrophic Lateral Sclerosis. Front. Mol. Neurosci..

[291] Martinelli A.H.S., Lopes F.C., John E.B.O., Carlini C.R., Ligabue-Braun R. (2019). Modulation of Disordered Proteins with a Focus on Neurodegenerative Diseases and Other Pathologies. Int. J. Mol. Sci..

[292] Armiento V., Spanopoulou A., Kapurniotu A. (2020). Peptide-Based Molecular Strategies To Interfere with Protein Misfolding, Aggregation, and Cell Degeneration. Angew. Chem. Int. Ed. Engl..

[293] Singh M., Singh B.K., Rai S.N., Singh P., Varshney R., Chaturvedi V.K., Vamanu E. (2021). Promising drug targets and associated therapeutic interventions in Parkinson’s disease. Neural Regen. Res..

[294] Eisele Y.S., Monteiro C., Fearns C., Encalada S.E., Wiseman R.L., Powers E.T., Kelly J.W. (2015). Targeting protein aggregation for the treatment of degenerative diseases. Nat. Rev. Drug Discov..

